# Burden, risk factors, and emerging microbiological trends of Gram-negative neonatal sepsis in Jordan: a retrospective cohort study

**DOI:** 10.1186/s12879-026-13529-7

**Published:** 2026-05-18

**Authors:** Eman F. Badran, Areej Sharaqa, Barakat Alsaqqa, Sanad T. Haddad, Hala Al-Jaberi, Loiy T. Algazo, Oraib Al-Smadi, Taimein Yacoub, Shahd Rihan, Enas Jaradat, Sara Al-Zayadneh, Bayan Shaheen

**Affiliations:** 1 Pediatrics Department, School of Medicine, The University of Jordan, Amman, 11942 Jordan; 2 Department of Neonatology, Princess Rahmah Teaching Hospital, Ministry of Health, Irbid, 21110 Jordan

**Keywords:** Neonatal sepsis, Gram-negative bacteria, Antimicrobial resistance, Jordan, Epidemiology

## Abstract

**Background:**

Neonatal sepsis (NS) is a major cause of morbidity and mortality, particularly in low- and middle-income countries. Gram-negative bacteria are increasingly predominant and associated with severe disease. In Jordan, data on Gram-negative NS are limited, highlighting the need for local evidence to guide early detection and treatment.

**Objective:**

To determine the incidence, risk factors, bacterial profile, and antimicrobial resistance patterns of Gram-negative NS in a tertiary Neonatal Intensive Care Unit (NICU) in Jordan.

**Methods:**

A retrospective cohort study reviewed 4,804 blood cultures from 3,429 neonates admitted to the NICU at Jordan University Hospital over six years. Clinical, maternal, and perinatal data were extracted from electronic health records. Descriptive statistics were used to summarize patient characteristics and microbiological findings, while regression analysis was used to identify risk factors for Gram-negative NS.

**Results:**

Among 4,172 blood cultures, 72 Gram-negative NS cases were identified, with a cumulative incidence of 9.7 per 1,000 NICU admissions. Extremely preterm neonates and those receiving early invasive interventions had higher risk (aOR 1.88–6.89), while elevated C-reactive protein and thrombocytopenia were independently associated. *Klebsiella pneumoniae* (30.6%) and *Escherichia coli* (22.2%) predominated. Multidrug resistance was observed in 34.7% of isolates, 23.6% were extended-spectrum β-lactamase producers, with high cephalosporin resistance and alarming meropenem resistance (33.3%).

**Conclusion:**

Multidrug-resistant Gram-negative pathogens remain the predominant cause of neonatal sepsis in this Jordanian tertiary NICU, with emerging resistance to last-line antibiotics, including carbapenems. These findings highlight the importance of ongoing surveillance and support future efforts in infection prevention and antibiotic stewardship.

**Supplementary Information:**

The online version contains supplementary material available at 10.1186/s12879-026-13529-7.

## Background

Neonatal sepsis (NS) is a leading cause of morbidity and mortality among neonates, posing a significant global health challenge, especially in low- and middle-income countries (LMICs) [1–2]. The incidence and mortality rates vary widely due to marked heterogeneity and regional differences. Globally, the estimated neonatal sepsis incidence is approximately 2,824 per 100,000 live births, with an overall mortality rate of around 17.6%. In LMICs, NS is estimated to cause approximately 550,000 deaths per year, accounting for about 15% of neonatal mortality [2–3]. NS is an illness caused by bloodstream infections of bacterial, fungal, or viral origin that affects newborn infants under 28 days of age [4–5]. It is classified according to chronology into early-onset, occurring within the first 3 days of life, and late-onset, which occurs from the fourth day of life [5].

Bacterial pathogens, including both Gram-positive and Gram-negative organisms, are the most commonly identified causes of neonatal sepsis [4]. In neonates, particularly preterm infants, bacterial sepsis is facilitated by an immature and functionally dysregulated immune system, impaired physiologic barrier function, limited maternal IgG transfer, and weakened mucosal defenses [6].

Gram-negative pathogens have increasingly emerged as major contributors to severe neonatal infections, particularly in LMIC settings [7–9]. Evidence suggests that Gram-negative sepsis is associated with higher mortality and worse clinical outcomes than Gram-positive infections, although outcomes are influenced by multiple clinical and microbiological factors [9]. Additionally, inflammatory markers such as C-reactive protein (CRP), Procalcitonin (PCT), and Tumor Necrosis Factor-Alpha (TNF-α) are notably elevated in cases of Gram-negative sepsis, highlighting the greater severity of its illness [8]. Moreover, rising multidrug resistance among Gram-negative organisms—including extended-spectrum beta-lactamase (ESBL) production—poses significant therapeutic challenges in neonatal intensive care units (NICUs), especially in resource-limited settings [10].

Risk factors for neonatal sepsis include maternal, perinatal, and neonatal factors [10]. Key maternal risk factors include chorioamnionitis, prolonged rupture of membranes (> 18 h), and maternal fever (> 38 °C). Important neonatal risk factors include prematurity, low birth weight, invasive procedures, and low Apgar scores [10].

Despite the substantial global burden of Gram-negative neonatal sepsis, comprehensive data from Jordan—including incidence, risk factors, bacteriological profiles, and antimicrobial resistance patterns—remain limited. This lack of local evidence restricts the development of context-specific empirical antibiotic protocols and infection control policies.

Given the increasing severity and antimicrobial resistance associated with Gram-negative infections, this retrospective cohort study aimed to determine the cumulative incidence and risk factors of early- versus late-onset Gram-negative sepsis among neonates admitted to the Neonatal Intensive Care Unit (NICU) at the University of Jordan Hospital (JUH), and to characterize the bacteriological profile and antimicrobial resistance patterns of the causative pathogens. The findings of this study provide local epidemiological data that support the development of context-specific therapeutic strategies and antimicrobial stewardship initiatives in Jordan and similar settings.

## Materials and methods

### Study design and population

This retrospective cohort study involved a detailed review and analysis of the electronic health records (EHRs) of all neonates admitted to the neonatal intensive care unit (NICU) of Jordan University Hospital (JUH) in Amman, Jordan, between March 2018 and April 2024. All neonates were followed from admission until discharge or death, neonates with major congenital anomalies or without blood cultures were excluded.

The unit of analysis was the blood culture result rather than the individual patient. Blood cultures were classified as positive or negative. The comparison group included blood culture episodes negative for sepsis from neonates with no confirmed infections at other sites (urinary tract, meningitis, pneumonia, or skin/soft tissue) to maintain a non-infected comparison group consistent with the study objective.

**Fig. 1:**
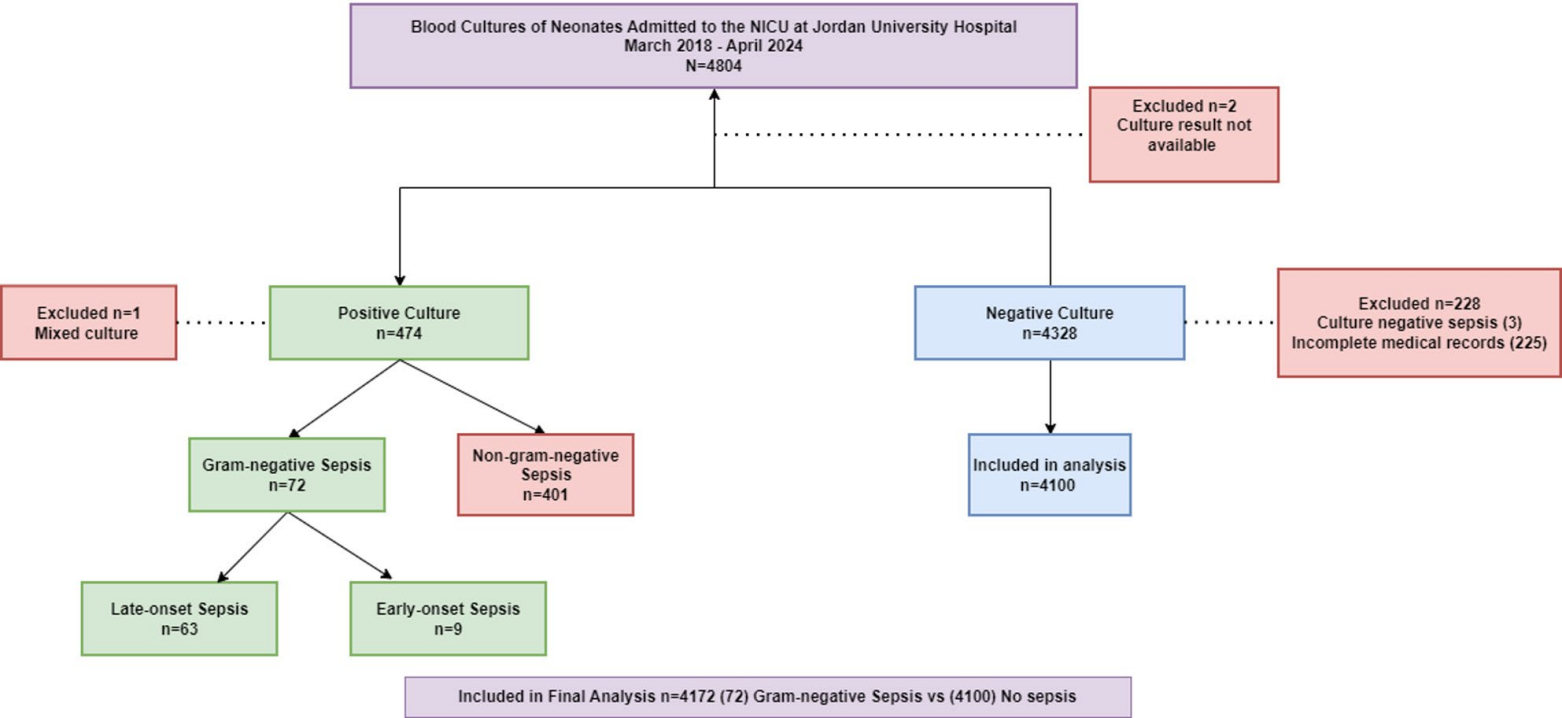
Flowchart of included and excluded study units

Mixed microbial growth; non-Gram-negative organisms (Gram-positive bacteria and fungi); culture-negative sepsis (defined as the presence of documented clinical signs of sepsis according to standardized NICU protocols and receipt of antibiotic therapy for ≥ 5 days despite negative blood cultures, with no alternative diagnosis identified); and samples with incomplete medical records were excluded from analysis. Gram-negative isolates were further classified as early-onset or late-onset sepsis. Full details of sample selection and exclusion are presented in Fig. 1.

### Study setting

The NICU at JUH is a tertiary-level referral center, with a total capacity of 34 beds, including four general care rooms and three isolation rooms. The general rooms include three rooms with seven beds each and one room with nine beds, while the isolation facilities consist of two single-patient rooms and one isolation room with a capacity of two beds. The unit admits around 1,000 neonates annually.

Infection control is maintained through a strict policy of hand washing, use of isolation rooms, and aseptic medication and total parenteral nutrition (TPN) preparation, and a regular surveillance system. The nurse-to-patient ratio is maintained at 1:3, with nursing staff working in three rotating shifts. This unit is fully equipped to provide care for critically ill and preterm infants, has implemented the Kaiser’s early-onset sepsis risk calculator guidelines [11] since 2015, and has been accredited as a Baby-Friendly Hospital since 2019.

### Data collection and measurements

Data were extracted from electronic health records (EHRs), and only complete, verified records were included. Collected data encompassed patient demographics (sex and age at sepsis diagnosis) and maternal and perinatal variables, including mode of delivery, gestational age, and birth weight. Clinical condition at birth was assessed using APGAR scores at 1 and 5 min, the need for resuscitation measures, and intubation. Feeding methods and other relevant clinical parameters were also recorded.

Laboratory markers analyzed included C-reactive protein (CRP) and complete blood count with differential, including platelet count. Values obtained at the time of initial clinical suspicion of sepsis were used, defined as measurements collected within 24 h before or after blood culture collection. When multiple measurements were available within this interval, the value closest in time to the blood culture collection was selected for analysis. Standard cutoffs were applied for all laboratory parameters based on established neonatal reference ranges, with complete definitions provided in Additional File 1 [12–19]. Key cutoffs included: anemia (hemoglobin < 13.5 g/dL) [13], leukopenia (white blood cell count < 5 × 10⁹/L) [4, 8], leukocytosis (> 25 × 10⁹/L) [4, 8], neutropenia (absolute neutrophil count < 1.5 × 10⁹/L) [14, 15], lymphopenia (absolute lymphocyte count < 2 × 10⁹/L) [15], elevated CRP (10–100 mg/L for increased; >100 mg/L for markedly increased) [16–18], and thrombocytopenia (platelet count < 150 × 10⁹/L) [18].

Neonatal Gram-negative sepsis was categorized as early-onset sepsis (EOS; culture-proven bloodstream infection within ≤ 72 h of life) or late-onset sepsis (LOS; culture-proven bloodstream infection occurring > 72 h of life). Key clinical variables included APGAR scores at 1 and 5 min, birth weight categories, and hematologic parameters (anemia and thrombocytopenia). Full definitions and categorizations of all study variables, including demographic, clinical, laboratory, microbiologic, and intervention variables, are provided in Additional file 1.

### Bacterial identification and antimicrobial susceptibility testing

Bacterial identification and antimicrobial susceptibility testing (AST) were performed using the VITEK 2 Compact system (bioMérieux, Marcy-l’Étoile, France). Gram-negative isolates were identified using the GN card. AST was conducted using the N417 AST card for Enterobacteriaceae and the N419 AST card for Pseudomonas species. For multidrug-resistant organisms (MDROs) requiring additional evaluation, the XN20 AST card was used as an extension panel. The VITEK 2 system employs colorimetric reagent cards and growth-based technology to generate quantitative susceptibility results, reported as susceptible, intermediate, or resistant; intermediate results were classified as resistant for conservative estimates. Multidrug resistance (MDR) was defined as acquired non-susceptibility to at least one agent in three or more antimicrobial categories, consistent with international standards [12]. Extended-spectrum beta-lactamase (ESBL) production was detected using the GN76 AST card and interpreted according to CLSI antimicrobial susceptibility testing guidelines [13]. All AST reporting followed CLSI standards [13] and national laboratory protocols in Jordan [20].

Culture 

### Culture withdrawal, diagnosis and treatment

At least 1 mL of blood is collected per culture, processed within two hours using the BACTEC BD system, and incubated for 5–7 days, with alerts issued within 48 h for positive results, prompting withdrawal of an additional 1 mL blood sample. Positive cultures undergo Gram staining, culturing, confirmatory identification, and AST using the VITEK-2 Compact system [12, 13, 20].

As per JUH NICU protocol regarding sepsis work up, blood cultures and empiric antibiotics are initiated in neonates < 32 weeks gestation, those with major sepsis risk factors, or showing clinical signs of sepsis, within 1 h of suspicion. First-line therapy typically includes Ampicillin plus an Aminoglycoside. Second-line therapy is Vancomycin and Meropenem, with the addition of Amikacin if the neonate is hemodynamically unstable. Antibiotics for confirmed Gram-negative infection are continued for 14–21 days (21 days for meningitis or if lumbar puncture could not be performed) and adjusted based on culture results. Therapy is stopped once neonates are clinically stable with negative culture and two consecutive CRP test result < 10 mg/dL 24 h apart.

### Unit of analysis and handling of repeated cultures

The unit of analysis in this study was the individual blood culture rather than the individual neonate. A total of 4,804 blood cultures were obtained from 3,429 neonates during the study period (mean 1.4 cultures per neonate), with the majority of neonates (78.5%) contributing only one culture during their NICU admission.

For the primary outcome of Gram-negative sepsis, 72 Gram-negative cultures were identified from 66 unique neonates. Six neonates contributed two Gram-negative cultures each, which represented repeat cultures within the same sepsis episode. Logistic regression analysis was performed at the culture level. We acknowledge that including multiple cultures from the same neonate may violate the independence assumption of standard regression.

### Statistical analysis

#### Description of study population at baseline

Data analysis was conducted using IBM SPSS Statistics (version 31.0.1.0; IBM Corp., Armonk, NY, USA). Continuous variables were summarized as mean ± standard deviation (SD) for normally distributed data, or as median (interquartile range) for non-normally distributed data. Categorical variables were presented as frequencies and percentages. For baseline comparison, participants were categorized into three groups: no sepsis (control group), EOS, and LOS. Comparisons of continuous variables across groups were performed using non-parametric tests (Kruskal-Wallis), given skewed distributions, while categorical variables were compared using the chi-square or Fisher’s exact test, as appropriate. A p-value of < 0.05 was considered statistically significant. Given the small number of EOS cases (*n* = 9), all EOS-specific analyses are descriptive and exploratory.

The cumulative incidence of Gram-negative neonatal sepsis was determined among all NICU admissions by dividing the number of neonates with culture-confirmed Gram-negative sepsis by the total number of NICU admissions, and expressing the result per 1,000 admissions. This measure reflects the overall burden of disease within the NICU population. The rate of Gram-negative neonatal sepsis among neonates who underwent blood culture testing was calculated by dividing the number of neonates with culture-confirmed Gram-negative sepsis by the number of neonates who had blood cultures obtained due to clinical suspicion or high-risk status as per NICU protocol, and expressing the result per 1,000 tested neonates. This approach aligns with standard epidemiologic practices for reporting healthcare-associated infections within defined at-risk populations [21].

#### Factors associated with gram-negative sepsis

Pooled multivariable logistic regression was performed to identify neonatal, clinical, and laboratory factors independently associated with Gram-negative sepsis (binary outcome: sepsis vs. no sepsis). Regression analyses combined early- and late-onset sepsis into a single outcome due to the small number of EOS cases (*n* = 9) to ensure model stability. For clinical interventions applied only to subsets of neonates (central line placement and intubation), analyses were restricted to participants who received the intervention, with timing relative to blood culture collection incorporated to ensure valid risk estimation. Results from these analyses are presented as odds ratios (ORs) with 95% confidence intervals (CIs).

#### Sensitivity analysis

Sensitivity analyses were performed to assess the robustness of key associations and address potential confounding by clinical interventions. The apparent protective effect of extreme prematurity in unadjusted analyses (OR 0.185) was hypothesized to reflect confounding, as extremely preterm neonates are more likely to receive interventions that increase sepsis risk. Separate logistic regression models were constructed for gestational age and platelet abnormalities, adjusting for central line placement, intubation, surgery, blood transfusions, and chest tube placement. All analyses used the same multiply imputed datasets and pooled estimates as the primary models. Adjusted odds ratios (aORs) with 95% confidence intervals were reported.

#### Bacteriological profile and resistance patterns

The bacterial profile was characterized using counts and percentages for all sepsis cases, and stratified by EOS and LOS. MDR and ESBL prevalence were reported similarly for each species. Antimicrobial resistance rates were calculated as the proportion of resistant isolates among all isolates tested for each antibiotic, regardless of species, to estimate overall resistance patterns. For species-specific resistance patterns (e.g., ESBL prevalence in *Klebsiella pneumoniae*), rates were calculated as the proportion of resistant isolates within that species. To ensure robustness, only antibiotics tested in ≥ 30 isolates were included, in accordance with the CLSI M39 guideline on cumulative antimicrobial susceptibility data [22]. Given the high observed meropenem resistance, species-specific analyses were performed to identify the main contributing organisms. All bacterial profiles, MDR and ESBL prevalence, and resistance rates were summarized and visualized in stacked column charts.

#### Data cleaning and handling of missing data

The extent of missing data was assessed prior to analysis. Multiple imputation was performed using fully conditional specification (FCS) with 20 imputations and a maximum of 10 iterations for convergence. Variables with 5–20% missingness, were imputed, whereas variables with < 5% missing data were analyzed without imputation, no variables had more than 20% missingness [23]. All imputed variables were categorical and were modeled using appropriate logistic or multinomial regression models within the FCS framework. Imputation was conducted under the assumption that data were missing at random and included all variables used in the analytical models. Results from the imputed datasets were combined using Rubin’s rules to produce pooled estimates. The reported odds ratios, confidence intervals, and p-values represent the combined results across all 20 imputed datasets, appropriately accounting for the uncertainty introduced by imputation. A two-sided p-value < 0.05 was considered statistically significant.

## Results

### Cumulative incidence of gram-negative sepsis in study population

During the study period, 6,788 neonates were admitted to the NICU, of whom 3,429 had blood cultures obtained. A total of 4,804 blood culture samples were collected, as multiple cultures could be obtained from the same neonate. Blood cultures were initially classified as positive (*n* = 474) or negative (*n* = 4,328). Two cultures were excluded due to unavailable results.

Among the culture-positive samples, one was excluded because of mixed microbial growth. The remaining positive cultures were categorized as Gram-negative (*n* = 72) and non-Gram-negative (*n* = 401), including Gram-positive bacteria and fungi, which were excluded from analysis. Gram-negative isolates were further classified into early-onset (*n* = 9) and late-onset sepsis (*n* = 63). Of the culture-negative samples, three were excluded as culture-negative sepsis, and an additional 225 samples were excluded due to incomplete medical records. The final analysis included 72 episodes of Gram-negative neonatal sepsis and 4,100 blood culture episodes classified as non-sepsis, defined as negative cultures in neonates without confirmed infections at other anatomical sites. In total, 632 blood culture samples were excluded. Full details are presented in Fig. 1.

Seventy-two Gram-negative isolates were identified from 66 unique neonates. Six neonates contributed two cultures each from the same sepsis episode. All 72 Gram-negative cultures were included in the regression analysis, and we acknowledge that the inclusion of repeat cultures may introduce residual within-neonate correlation.

Among the 3,429 neonates from whom blood cultures were obtained, 66 tested positive for Gram-negative pathogens, corresponding to a rate of 19.3 cases per 1,000 tested neonates. Based on a total of 6,788 NICU admissions, the cumulative incidence of culture-confirmed Gram-negative sepsis was 9.7 cases per 1,000 NICU admission.

### Characteristics of study population at baseline

Baseline characteristics of the study participants are summarized in Table 1 (available at end of document). Among the 4,172 blood culture samples included in the analysis, 98.3% were in the no-sepsis group, and 1.7% were in the Gram-negative sepsis group, of which 87.5% had LOS and 12.5% had EOS.

**Table 1:** Baseline clinical characteristics, lab findings, interventions, and outcomes of neonatal blood cultures by sepsis status (N* = 4,172)

Characteristic	No Sepsis *n* = 4100	LOS *n* = 63	EOS *n* = 9
	*n* (%)	*n* (%)	*n* (%)
**Gender**			***P*** ** = 0.603**
Male	2414 (58.9)	39 (61.9)	4 (44.4)
Female	1686 (41.1)	24 (38.1)	5 (55.6)
**Mean gestational age (weeks) ± SD**	1.54 ± 1.306		
**Gestational age (weeks)**			***P*** ** < 0.001**
Extremely preterm < 28 weeks	235(5.9)	11(19.0)	4(50.0)
Very Preterm 28–32 weeks	816(20.6)	27(46.6)	1(12.5)
Moderate to late preterm 33–36 weeks	1409(35.5)	16(27.6)	1(12.5)
Term (≥ 37 weeks)	1504 (37.9)	4 (6.9)	2 (25.0)
**Mode of delivery**			***P*** ** = 0.004**
Vaginal	989 (24.1)	10 (15.9)	6 (66.7)
Cesarean	3111 (75.9)	53 (84.1)	3 (33.3)
**Mean birth weight (BW) ± SD**	1.38 ± 1.381		
**Birth weight categories (BW) (g)**			***P*** ** < 0.001**
Extremely low BW (ELBW) < 1000 g	264 (6.4)	14 (23.3)	2 (25.0)
Very low BW (VLBW) 1000–1499 g	526 (12.8)	20 (33.3)	2 (25)
Low BW (LBW) 1500–2499 g	1355 (33.0)	17 (28.3)	3 (37.5)
Normal BW (NBW) 2500–3999 g	1894 (46.2)	9 (15.0)	1 (12.5)
High BW (HBW) 4000–4500 g	54 (1.3)	0 (0.0)	0 (0.0)
Very high BW (VHBW) > 4500 g	7 (0.2)	0 (0.0)	0 (0.0)
**Apgar Score at 1 min**			***P*** ** = 0.026**
Severe distress ≤ 3	146(4.0)	7(12.7)	0 (0.0)
Moderate difficulty 4–6	180(4.9)	3(5.5)	0 (0.0)
Assuring ≥ 7	3338(91.1)	45(81.8)	4(100.0)
**Apgar Score at 5 min**			***P*** ** = 0.084**
Severe distress ≤ 3	12(0.3)	1(1.8)	0 (0.0)
Moderate difficulty 4–6	61(1.7)	3(5.5)	0 (0.0)
Assuring ≥ 7	3588 (98))	51(92.7)	4 (100.0)
Indices at suspension of Clinical sepsis**			
** C-reactive protein (CRP) (mg/L)**			***P*** ** < 0.001**
Increased: CRP 10–100 mg/L	642 (16.3)	34 (56.7)	4 (57.1)
Markedly Increased: CRP > 100 mg/L	30 (0.8)	16 (26.7)	0 (0.0)
** Hemoglobin (Hb) (g/dL)**			***P*** ** < 0.001**
Anemia: Hb < 13.5 g/dL	812(79.4)	40(70.2)	0 (0.0)
** White Blood Cell Count (WBC) (×10⁹/L)**			***P*** ** < 0.001**
Leukopenia: WBC < 5 × 10⁹/L	135(3.4)	13(22.8)	1 (11.1)
Leukocytosis: WBC > 25 × 10⁹/L	295(7.5)	3(5.3)	3 (33.3)
** Absolute Neutrophil Count (ANC) (×10⁹/L)**			***P*** ** < 0.001**
Neutropenia: ANC < 1.5 × 10⁹/L	142(3.6)	12(21.1)	1(11.1)
** Absolute Lymphocyte Count (ALC) (×10⁹/L)**			***P*** ** < 0.001**
Lymphopenia: ALC < 2 × 10⁹/L	398(10.1)	19(33.3)	3 (33.3)
** Platelet Count (Plt) (×10⁹/L)**			***P*** ** < 0.001**
Thrombocytopenia: Plt < 150 × 10⁹/L	577 (14.7)	31 (54.4)	3 (33.3)
Thrombocytosis: Plt > 450 × 10⁹/L	253 (6.4)	1 (1.8)	0 (0.0)
**Feeding Type**			***P*** ** = 0.336**
Mother’s milk	2245 (65.7)	38 (73.1)	4 (66.7)
Formula	122 (3.6)	3 (5.8)	0 (0.0)
Mixed feeding (Mother’s milk + formula)	1040 (30.5)	10 (19.2)	2 (33.3)
Donor milk	1(0.0)	0 (0.0)	0 (0.0)
Mother and donor milk	6 (0.2)	1 (1.9)	0 (0.0)
Formula and donor milk	1(0.0)	0 (0.0)	0 (0.0)
**Umbilical Venous Catheter (UVC) Insertion**			***P*** ** = 0.453**
	773 (18.9)	24 (38.1)	4 (44.4)
Central Line (CL) Placement **			***P*** ** < 0.001**
	106 (2.6)	7 (11.1)	1 (11.1)
**Intubation at any time over the course of hospital stay**			***P*** ** < 0.001**
	651 (15.9)	26 (41.3)	5 (55.6)
**Blood Transfusion at any time over the course of hospital stay**			***P*** ** < 0.001**
	872 (21.3)	38 (60.3)	4 (44.4)
**Surgery Done at any time over the course of hospital stay**			***P*** ** < 0.001**
	432 (10.5)	20 (31.7)	1 (11.1)
**Chest Tube Insertion at any time over the course of hospital stay**			***P*** ** = 0.378**
	108 (2.6)	0 (0.0)	0 (0.0)
**Abdominal Paracentesis at any time over the course of hospital stay**			***P*** ** = 0.983**
	2 (0.0)	0 (0.0)	0 (0.0)
**Cardiopulmonary Resuscitation at any time over the course of hospital stay (CPR)**			***P*** ** < 0.001**
	255 (6.2)	19 (30.2)	3 (33.3)
**Retinopathy of Prematurity (ROP)**			***P*** ** = 0.004**
	43 (1.0)	2 (3.2)	1 (11.1)
**Death**			***P*** ** < 0.001**
	305 (7.4)	24 (38.1)	2 (22.2)
**Length of Stay Median (IQR)** *n* = 4172			***P*** ** < 0.001**
**Length of Stay (Median)**	12	45	27
**Inter Quartile Range (IQR)**	(6–35)	(28–66)	(12–84)
**Median age at CL insertion (days) (IQR)** *n* = 114			***P*** ** = 0.706**
**Age at CL insertion (Median)**	6	2	-
**Inter Quartile Range (IQR)**	(2–20)	(1-8.5)	-
**Median age at Intubation (days) (IQR)** *n* = 682			***P*** ** = 0.097**
**Median age at intubation**	0	0	1
**Inter Quartile Range (IQR)**	(0–1)	(0–0)	(0–2)

*N indicates a total number of blood culture samples carried out at starting point; certain patients provided multiple blood cultures. Analyses have been done at the blood culture level**.**

** Central Line (CL) Insertion: include peripherally inserted central catheters (PICC Lines) and Broviac central lines

### Demographic and perinatal descriptions

The overall sex distribution was similar across study groups. Gestational age was lower in sepsis groups, with very preterm neonates (28–32 weeks) more commonly affected by LOS (46.6%) and extremely preterm neonates (born prior to 28 weeks) accounted for 50% of early-onset sepsis cases (*p* < 0.001). The mode of delivery varied considerably among groups (*p* = 0.004), cesarean delivery was more frequent in LOS (84.1%) than in controls (75.9%), whereas most EOS cases (66.7%) were delivered vaginally.

### Neonatal birthweight and status at birth

The distribution of birth weights varied significantly among groups (*p* < 0.001), the majority of infants in the no-sepsis group (46.2%) were of normal birth weight, while sepsis cases showed an overrepresentation of extremely low birth weight (ELBW) and very low (VLBW). ELBW accounted for (25%) of EOS and (23.3%) of LOS, while VLBW comprised (25%) and (33.3%) of cases, respectively. The rate of abnormal Apgar scores at 1 min was significantly higher in the LOS group (12.7%) compared to the no-sepsis group (4.0%, *p* = 0.026), while no cases (0%) were observed in the EOS group.

### Laboratory results

During the time of suspected clinical sepsis, inflammatory and hematologic indices showed significant differences among groups (all *p* < 0.001). Markedly elevated CRP (> 100 mg/L) was present in 26.7% of LOS cases but was rare (0.8%) in the no-sepsis group and absent in EOS. Hematologic abnormalities, particularly thrombocytopenia (LOS: 54.4%, EOS: 33.3%, No-Sepsis: 14.7%) and leukopenia (LOS: 22.8%, EOS: 11.1%, No-Sepsis: 3.4%), were significantly more prevalent in septic cases.

### Clinical procedures and interventions

Feeding type distributions were comparable across groups (*p* = 0.336), with exclusive breastfeeding (mother’s milk) being the most common (LOS:73.1%, EOS:66.7%, No sepsis:65.7%). In contrast, invasive interventions were significantly more frequent in sepsis groups, including intubation (41.3% LOS, 55.6% EOS), blood transfusion (60.3% LOS), and central line placement (11.1% in both sepsis groups) (all *p* < 0.001).

### Outcome results

Neonates with sepsis showed markedly worse outcomes. Mortality rates were higher in the LOS (38.1%) and EOS (22.2%) groups relative to the no-sepsis group (7.4%) (*p* < 0.001). LOS and EOS were also associated with longer hospital stays, with median durations of 45 days and 27 days, respectively, in comparison to 12 days in neonates without sepsis (*p* < 0.001), reflecting the high-acuity nature of neonates admitted to our tertiary NICU. Many of these neonates were preterm and required prolonged hospitalization for underlying conditions. Retinopathy of prematurity was more prevalent among sepsis cases (*p* = 0.004), as was the need for cardiopulmonary resuscitation (*p* < 0.001).

### Factors associated with gram-negative sepsis

Pooled multivariable logistic regression was performed to identify neonatal, clinical, and laboratory factors independently associated with Gram-negative sepsis (binary outcome: sepsis vs. no sepsis).

Analyses are presented across multiple tables to preserve clarity and interpretability. Table 2 shows the primary pooled logistic regression for the full cohort. Model performance metrics and sensitivity analyses adjusting for clinical interventions (gestational age and platelet abnormalities) are provided in Additional files 4–5. Readers are referred to the supplementary files for details of these analyses.

The results of the primary analysis are summarized in Table 2, estimates were pooled from 20 multiply imputed datasets using Rubin’s rules, and reported odds ratios, confidence intervals, and p-values reflect these pooled results.

### Demographic and perinatal factors

In the unadjusted analysis, gestational age was significantly associated with sepsis. Extremely preterm neonates had lower odds of sepsis compared with term infants (OR = 0.185, 95% CI 0.51–0.674, *p* = 0.011). This unexpected finding is likely explained by confounding, as extremely preterm infants have a higher baseline risk of sepsis and are more likely to receive intensive clinical interventions. To address these potential confounders, a sensitivity analysis was conducted adjusting for relevant factors.

### Laboratory parameters

Among laboratory parameters, elevated C-reactive protein was strongly associated with sepsis, with increased CRP (OR = 14.808, 95% CI 6.415–34.192, *p* < 0.001) and markedly increased CRP (OR = 174.208, 95% CI 47.603-637.536, *p* < 0.001) serving as key diagnostic and prognostic markers. Thrombocytopenia was similarly associated with sepsis (OR = 2.564, 95% CI 1.252–5.253, *p* = 0.010), reflecting early platelet consumption and coagulation disturbances. Both CRP and platelet count were measured at the time of clinical suspicion, representing current physiological status rather than baseline risk, and thus function as indicators of disease severity rather than predisposing factors.

### Clinical procedures and interventions

#### Timing of Central Line (CL) placement

Neonates who had a central line inserted after the blood culture collection were associated with lower odds of Gram-negative sepsis compared with those who underwent the intervention before culture (OR 0.51, 95% CI 0.35–0.76, *p* < 0.001). However, age at central line placement per one-day increase was not significantly associated with sepsis (*p* = 0.906).

#### Timing of intubation

Similarly, neonates intubated after blood culture collection were associated with lower odds of Gram-negative sepsis compared with those intubated before culture (OR 0.39, 95% CI 0.28–0.53, *p* < 0.001). However, age at intubation (per one-day increase) was not significantly associated with sepsis (*p* = 0.319).

These findings indicate that neonates who underwent interventions such as central line placement or intubation before blood culture collection were more likely to have Gram-negative sepsis, whereas interventions after culture collection were associated with lower odds. These associations should be interpreted cautiously, as the timing of interventions may reflect differences in underlying clinical severity and treatment context rather than a causal effect of the procedures themselves.

Other variables, including gender, birth weight, mode of delivery, Apgar scores, all clinical interventions, as well as NICU length of stay, were not significantly associated with sepsis. Full regression results are provided in Additional file 2.

**Table 2:** Pooled logistic regression analysis of factors associated with neonatal Gram-negative sepsis (Sepsis vs. No sepsis)

Variable	Category (Reference)	OR	95% CI	*P* value
Gestational Age	Extremely preterm vs. Term	0.185	0.051–0.674	**0.011**
C-Reactive Protein (CRP)	Increased vs. Normal	14.808	6.415–34.192	**< 0.001**
	markedly increased vs. Normal	174.208	47.603-637.536	**< 0.001**
Platelet Count (Plt)*	Thrombocytopenia vs. Normal	2.564	1.252–5.253	**0.010**
Timing of CL Placement Relative to Blood Culture**	After vs. Before culture	0.51	0.35–0.76	**< 0.001**
Age at CL Placement (Days)**	Per 1-day increase	0.999	0.99–1.01	0.906
Timing of Intubation Relative to Blood Culture**	After vs. Before culture	0.39	0.28–0.53	**< 0.001**
Age at Intubation (Days)**	Per 1-day increase	1.00	0.999–1.003	0.319

**Analyzed only in the subset of neonates who received the intervention (central line: *n* = 114; intubation: *n* = 682)

Pooled estimates are from 20 multiply imputed datasets combined using Rubin’s rules; odds ratios, confidence intervals, and p-values reflect these pooled results

### Model performance

Model fit was assessed to evaluate the overall performance, calibration, and reliability of the logistic regression analyses. As summarized in Additional file 4, all models exhibited adequate calibration according to Hosmer-Lemeshow goodness-of-fit tests (*p* > 0.05), indicating accurate prediction of observed outcomes.

The logistic regression model applied to the full cohort demonstrated consistent overall performance, with predictors significantly distinguishing sepsis cases (Omnibus test *p* < 0.001). Furthermore, the model explained a moderate portion of the variability in sepsis outcomes (Nagelkerke R²), indicating meaningful explanatory power.

In contrast, models restricted to subgroups of neonates who received a central line or intubation showed adequate calibration but poor overall predictive performance. In these subgroups, the predictors were not statistically significant as a set (non-significant Omnibus test) and explained very little of the variability in sepsis (very low pseudo R² values). This is likely due to the smaller sample sizes and reduced outcome variation within these specific groups, rather than a flaw in the modeling approach.

Therefore, while the subgroup analyses offer exploratory and supplementary insights, the complete cohort model remains the primary and most comprehensive analysis for identifying factors associated with Gram-negative neonatal sepsis.

### Sensitivity analysis

To assess the robustness of key findings and potential confounding by clinical interventions, separate sensitivity analyses were conducted for gestational age and platelet abnormalities.

#### Gestational age

After the unadjusted analysis suggested lower odds of sepsis among extremely preterm infants, a sensitivity analysis adjusting for clinical interventions showed that gestational age was strongly associated with sepsis risk across all categories. Extremely preterm infants had the highest odds (aOR 6.89, 95% CI 5.49–8.65), followed by very preterm (aOR 5.30, 95% CI 4.33–6.49) and moderate-late preterm infants (aOR 2.97, 95% CI 2.42–3.64). Clinical interventions including central line placement, intubation, surgery, and blood transfusions were independently associated with increased sepsis risk (aORs 1.88–2.31).

These results indicate that the protective effect of extreme prematurity in the unadjusted analysis was due to confounding by interventions, and in fact extremely preterm infants have a substantially higher risk of Gram-negative sepsis. Full results are presented in Additional file 5.

#### Platelet count

To assess the robustness of the association between thrombocytopenia and Gram-negative sepsis, we performed a sensitivity analysis adjusting for clinical interventions. Thrombocytopenia remained strongly associated with sepsis (aOR 4.37, 95% CI 3.91–4.89), central line placement, intubation, and blood transfusions were independently associated with increased sepsis risk (aORs 1.95–2.62). These findings reinforce that thrombocytopenia is a reliable diagnostic and prognostic marker of Gram-negative sepsis. Full results are shown in Additional File 5.

### Microbial profile and timing of sepsis

**Fig. 2:**
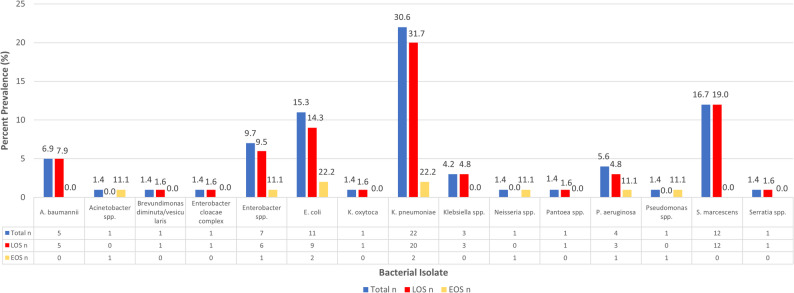
Distribution of Gram-negative isolates in total, early-onset (EOS), and late-onset (LOS) neonatal sepsis. *Klebsiella pneumoniae* and *Escherichia coli* were most common in EOS, while *Klebsiella pneumoniae*, *Serratia marcescens*, and *Escherichia coli* predominated in LOS. Some organisms were isolated exclusively in LOS (*Serratia marcescens*, *Acinetobacter baumannii*, *Klebsiella* spp.) while other in EOS (*Acinetobacter* spp., *Pseudomonas* spp., *Neisseria* spp.), results are presented as counts and percentages

Among all Gram-negative sepsis cases (*N* = 72), *Klebsiella pneumoniae* was the most frequently isolated organism, accounting for (*n* = 22, 30.6%) of cases (Fig. 2). *Escherichia coli* and *K. pneumoniae* were the most common organisms identified in EOS, each accounting for 22.2% of cases (44.4% combined). In LOS, *K. pneumoniae* was the predominant organism (31.7%), followed by *Serratia marcescens* (19.0%) and *E. coli* (14.3%). Overall, *K. pneumoniae* and *E. coli* were more frequently associated with LOS than EOS, with (90.9%) and (81.8%) of isolates occurring in LOS, respectively. *Pseudomonas aeruginosa* and *Enterobacter* spp. also showed higher association with LOS, accounting for (75%) and (85.7%) of cases, respectively.

Several organisms were isolated exclusively in LOS (100% prevalence), including *S. marcescens*,* Acinetobacter baumannii*, and *Klebsiella* spp., as well as single isolates of *Enterobacter cloacae* complex, *Brevundimonas diminuta/vesicularis*, *Klebsiella oxytoca*, *Serratia* spp., and *Pantoea* spp. (Fig. 2). In contrast, *Acinetobacter* spp., *Pseudomonas* spp., and *Neisseria* spp. were isolated exclusively in EOS.

For organisms identified only to the genus level (referred to as “species”), further species-level identification was not available, due to the limited supplies of VITEK system materials thus manual identification was done.

### Prevalence of MDR and ESBL-producing gram-negative bacteria

**Fig. 3:**
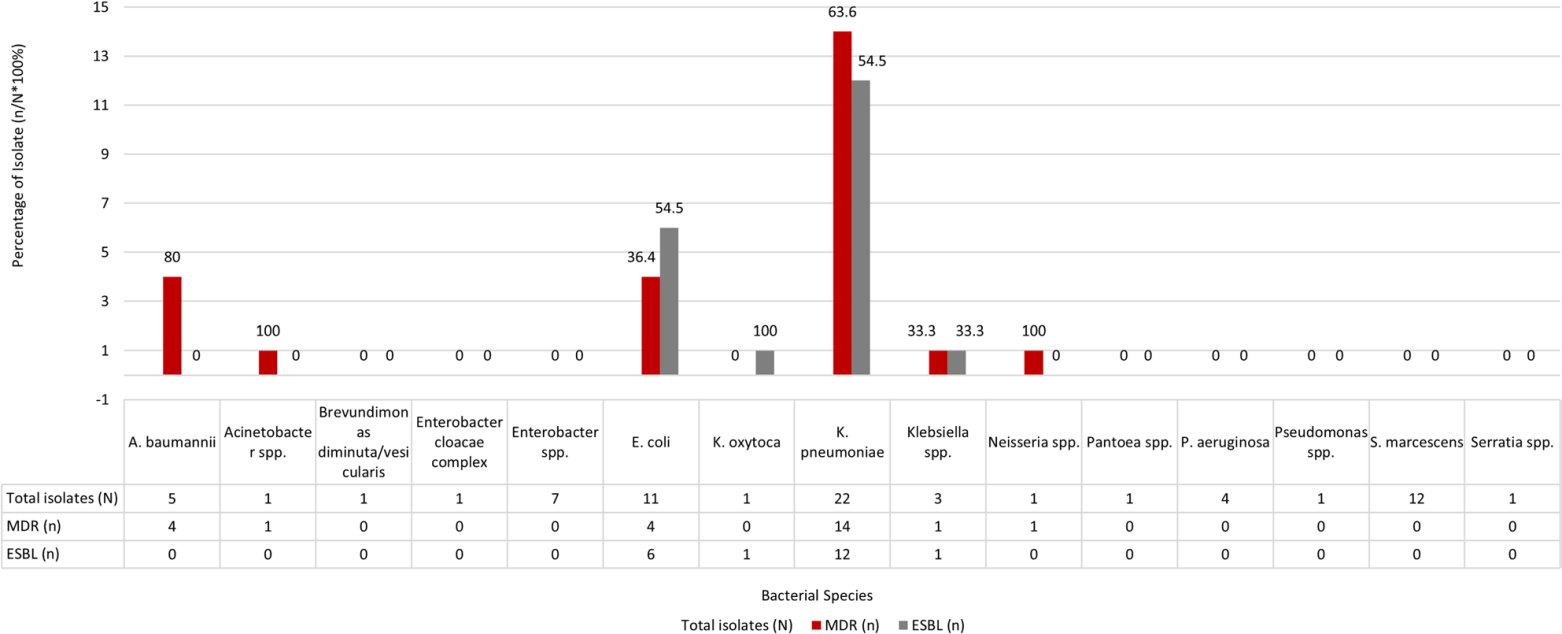
Distribution of multidrug-resistant (MDR) and extended-spectrum beta-lactamase (ESBL), presented as counts and percentages per species**. ***Klebsiella pneumoniae* and *Escherichia coli* exhibited the highest MDR (63.6% and 36.4%, respectively) and ESBL rates (both 54.5%), while other species showed lower prevalence of resistance

Among the 72 Gram-negative isolates obtained, multidrug-resistant organisms accounted for 34.7% (*n* = 25), with *K. pneumoniae* exhibiting the highest proportion of MDR isolates (63.6% within the species). Moreover, 17 (23.6%) of the isolates were confirmed to be ESBL producers, among these *K. pneumoniae* and *E. coli* were the most frequently identified, each accounting for 54.5% of ESBL isolates within their respective species (Fig. 3). Other Gram-negative species exhibited lower MDR and ESBL prevalence.

### Antibiotic resistance patterns

**Fig. 4:**
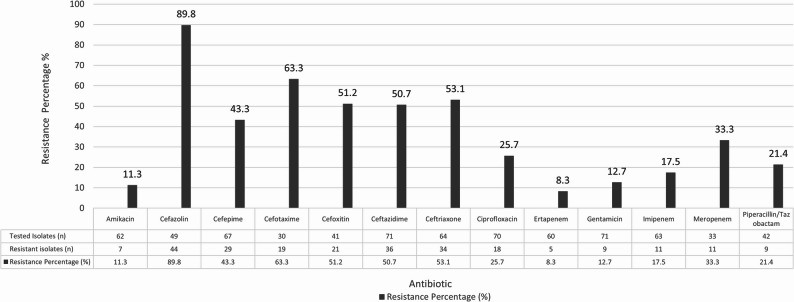
Overall antibiotic resistance patterns, restricted to agents evaluated in ≥ 30 isolates. Bars show the percentage of resistant isolates, with counts of tested and resistant isolates indicated below each antibiotic

Analysis of overall resistance patterns of the bacterial isolates revealed a high prevalence of resistance to beta-lactam antibiotics, particularly cephalosporins (Fig. 4). Cefazolin exhibited the highest resistance (89.3%), followed by Cefotaxime (63.3%), Ceftriaxone (53.1%), Cefoxitin (51.2%), and Ceftazidime (50.7%). Piperacillin/Tazobactam, a penicillin, showed notable resistance at 21.4%, while among carbapenems, Meropenem exhibited 33.3% resistance. Resistance was also observed in non-beta-lactam antibiotics, including Trimethoprim/ Sulfamethoxazole (53.1%) and Ciprofloxacin (25.7%). Species-specific analysis of meropenem resistance revealed that K. pneumoniae contributed most (60% of resistant isolates), followed by S. marcescens (20%) and A. baumannii (20%).

## Discussion

### The burden of gram-negative neonatal sepsis

Within this tertiary NICU cohort in Jordan, the cumulative incidence of culture-confirmed Gram-negative neonatal sepsis among all admissions was 9.7 per 1,000, whereas the rate among neonates who underwent blood culture testing was 19.3 per 1,000 tested neonates. Late-onset sepsis predominated, accounting for 87.5% of cases versus 12.5% for early-onset sepsis. These findings are consistent with previous reports from Jordan, which identified Gram-negative organisms as the predominant pathogens in neonatal late-onset sepsis [20, 24–25].

These findings must be interpreted within the broader LMIC context, where the burden of Gram-negative neonatal sepsis is substantially higher than in high-income countries [3, 10, 26–28]. This epidemiological gap carries a substantial human burden: while the global incidence of neonatal Gram-negative sepsis is roughly 2 per 1,000 live births, it rises to 4.35 per 1,000 live births in LMICs, reflecting a more than twofold increased risk for neonates in these settings [27]. Community-based cohort studies from sub-Saharan Africa and Southeast Asia estimate 6.5–15.2 per 1,000 live births for culture-confirmed severe bacterial infections, with Gram-negative pathogens responsible for roughly half of cases [28]. This incidence in our NICU is therefore consistent with the established, high regional burden observed across LMICs.

Clinically, this high incidence carries severe consequences. In this cohort, mortality among septic neonates was substantially higher than in non-septic neonates, with the highest rates observed in LOS (38.1%), followed by EOS (22.2%), and lowest among neonates without sepsis (7.4%) (*p* < 0.001). Median NICU stays were 45 days for LOS, 27 days for EOS, and 12 days for the non-sepsis group (IQR: 6–35), reflecting the high-acuity, referral nature of our NICU and baseline complexity of admitted neonates. Longer stays in septic neonates indicate the additional burden of infection. Gram-negative sepsis was also associated with higher rates of complications, including retinopathy of prematurity (ROP) and the need for cardiopulmonary resuscitation (*p* = 0.004 and *p* < 0.001, respectively). Residual confounding by baseline illness may remain despite adjustment for measured covariates. These findings align with international data indicating that LOS independently elevates mortality and prolongs hospitalization, particularly in cases of Gram-negative infection [29, 30].

The increased severity associated with LOS arises from its distinct etiology [20, 29–32]. Unlike the common vertical transmission of EOS, LOS frequently involves virulent, resistant organisms that can delay effective treatment, is typically healthcare-associated, and arises after prolonged NICU exposure [20, 30]. National data from Jordan further reinforce that Gram-negative pathogens predominate in LOS and are linked to higher mortality and severe disease markers [31, 32].

Ultimately, the cumulative burden of NICU exposures including prolonged hospitalization, invasive procedures, mechanical ventilation, and extended parenteral nutrition, likely contributes to hemodynamic instability and the increased need for CPR observed in septic neonates [29–32]. The higher ROP frequency observed among septic neonates in this cohort is likewise consistent with prior evidence identifying neonatal sepsis as a significant risk factor for ROP, particularly in cases of recurrent or late-onset infection [33, 34].

Therefore, within our setting, Gram-negative neonatal sepsis emerges as a major contributor to NICU morbidity and mortality.

### Factors associated with gram-negative neonatal sepsis

In this cohort, the risk of Gram-negative neonatal sepsis followed a clear gestational age-dependent gradient: extremely preterm neonates had the highest risk (aOR 6.89), followed by very preterm and moderate-to-late preterm neonates. International studies consistently identify prematurity as a significant risk factor for neonatal sepsis overall, with reported odds ratios ranging from 1.9 to 3.8 for neonates born before 37 weeks’ gestation [35–37]. Our findings align with evidence directly linking lower gestational age to an increased risk of Gram-negative infection and worse outcomes, including higher mortality from pathogens like *E. coli* [38, 39]. These consistent findings are rooted in the pathophysiology of prematurity; preterm infants have a 3-10-fold higher incidence of infection compared to term infants, largely due to immune immaturity, reduced transplacental maternal IgG, and increased exposure to invasive procedures [38–40]. These factors collectively explain the increased susceptibility of preterm neonates to severe Gram-negative infections and their associated worse outcomes.

In the present study, invasive clinical interventions were significantly associated with Gram-negative sepsis, particularly when performed prior to blood culture collection (central line placement: OR 0.51, 95% CI 0.35–0.76, *p* < 0.001; intubation: OR 0.39, 95% CI 0.28–0.53, *p* < 0.001). While device use such as intubation and central venous catheter placement is well-established as a risk factor for healthcare-associated bloodstream infections [14, 40, 41], these observed associations should be interpreted cautiously. The higher likelihood of sepsis observed following early interventions may be explained by disruption of primary host defenses, impaired microbial clearance, or facilitation of bloodstream invasion [16], whereas reductions in central-line use or duration have been associated with lower rates of culture-proven neonatal sepsis [42, 43]. Given the retrospective design, the timing of interventions relative to culture collection may reflect underlying illness severity or clinical indication rather than a direct causal effect. Neonates requiring early invasive support are often more critically ill and therefore inherently at higher risk of infection, whereas procedures performed after clinical suspicion of sepsis likely represent therapeutic response rather than a protective effect. Clinical severity at NICU admission is itself an independent risk factor for infection and frequently necessitates such interventions [44]. To mitigate infection risk, particularly in resource-limited settings, structured infection prevention strategies should be implemented, including World Health Organization (WHO) infection prevention frameworks and Central line-associated bloodstream infection (CLABSI) prevention protocols with strict aseptic technique, barrier precautions, proper site care, and minimization of catheter duration [41].

This study revealed strong associations between Gram-negative sepsis and laboratory markers, particularly CRP and thrombocytopenia. Elevated CRP, an acute-phase reactant synthesized by hepatocytes in response to pro-inflammatory cytokines, particularly interleukin-6 reflects systemic inflammatory activation during bacterial infection [4, 6, 7]. CRP levels typically rise within hours of infection and correlate with both the presence and severity of neonatal sepsis, serving as a supportive diagnostic and prognostic marker rather than a pre-existing risk factor [41, 45]. Thrombocytopenia is also commonly reported and independently associated with Gram-negative infections [38, 39, 44]. In these cases, systemic inflammation triggers increased platelet consumption and aggregation, sequestration in the microvasculature, bone marrow suppression by inflammatory cytokines, and immune-mediated platelet destruction. As such, thrombocytopenia serves as a diagnostic and prognostic marker, reflecting sepsis severity, coagulation disturbances, and endothelial injury [45]. Severe thrombocytopenia (platelets ≤ 50 × 10⁹/L; neonatal reference range 150–450 × 10⁹/L) occurs more frequently in Gram-negative sepsis than in Gram-positive infections, strongly predicting mortality and major bleeding complications, and demonstrates high diagnostic sensitivity (92.1%) when combined with other biomarkers [45].

Overall, these findings highlight that Gram-negative sepsis in preterm and critically ill neonates arises from inherent biological vulnerability and modifiable healthcare exposures, particularly in resource-limited NICUs.

### Bacterial etiology

Among the 72 Gram-negative sepsis isolates in this cohort, *Klebsiella pneumoniae* was the most prevalent (30.6%), followed by *Serratia marcescens* (16.7%) and *Escherichia coli* (15.3%). This distribution aligns with established national patterns in Jordan. For instance, Abdelfattah et al. similarly reported *E. coli* (17%) and *K. pneumoniae* (14.5%) as the most frequent isolates [24], whereas Yusef et al. highlight *Acinetobacter baumannii* (27%) and *K. pneumoniae* (22%) as predominant [46]. The significant predominant burden of *K. pneumoniae*, particularly in LOS, was highlighted by Khassawneh et al., where it accounted for 54.4% of Gram-negative cases, 61.1% as LOS [32]. Although a lower proportion was observed in our cohort (31.7% of LOS), this finding still reinforces its role as a major nosocomial pathogen in Jordanian NICUs.

A comparison with regional studies reveals notable differences and consistencies in pathogen patterns. For example, while Almudeer et al. identified *E. coli* as the predominant cause of EOS in Saudi Arabia (29% of all isolates), especially among very low birth weight neonates [47], our Jordanian cohort found an equal representation of *E. coli* and *K. pneumoniae* in EOS cases (22.2% each). However, both pathogens showed a stronger association with LOS in our setting, with *K. pneumoniae* accounting for 31.7% and *E. coli* for 14.3% of LOS cases, this aligns with a broader Gulf-region profile reported by Hammoud et al., where *E. coli*, *Acinetobacter* spp., and *Pseudomonas* spp. were frequently observed as causes of LOS [48].

On a global level, a meta-analysis of 88 studies confirms *Klebsiella* spp. as the leading Gram-negative cause of neonatal sepsis (~ 38% of cases), with the highest burden in LMICs, particularly Africa [10]. Overall, the consistent predominance of *K. pneumoniae* and *E. coli* in Jordan, regionally, and globally, confirms their standing as critical targets for neonatal care. This highlights the importance of ongoing surveillance, antimicrobial stewardship, and enhanced infection control measures to limit the spread of resistant Gram-negative organisms in neonatal settings.

### Antibiotic resistance patterns

Antibiotic resistance among Gram-negative neonatal sepsis isolates in the present cohort was substantial, MDR organisms accounted for over one third of isolates (34.7%), while ESBL-producers comprised nearly a quarter (23.6%). *K. pneumoniae* was the most concerning pathogen, exhibiting the highest rate of MDR, and together with *E. coli* accounted for most of ESBL-producing isolates. This distribution is consistent with prior reports from Jordanian NICUs, in which *Klebsiella* spp. were among the most frequent, highly resistant pathogens associated with severe clinical outcomes [25, 31, 46]. A local study by Al-Lawama et al. from 2014 found that more than half of Gram-negative neonatal sepsis isolates were resistant to third-generation cephalosporins, with *K. pneumoniae* being the predominant MDR pathogen [25] Similarly, Yusef et al. confirmed a national high burden of MDR Gram-negative organisms in neonatal sepsis, with *Klebsiella spp.* and *E. coli* accounting for the majority of resistant isolates [46].

Resistance patterns observed in this study were dominated by β-lactam resistance, particularly to cephalosporins. Resistance rates were highest for cefazolin (89.3%), followed by cefotaxime (63.3%), ceftriaxone (53.1%), cefoxitin (51.2%), and ceftazidime (50.7%), reflecting widespread ESBL activity. Comparable cephalosporin resistance rates have been reported in Jordanian NICUs, where resistance to third-generation cephalosporins among Gram-negative neonatal isolates ranged between 50% and 70% in earlier cohorts [25, 46]. Resistance to piperacillin/tazobactam in the present study (21.4%) is also consistent with regional data reporting resistance rates of approximately 20–30% among neonatal Gram-negative isolates [20, 49]. Of particular concern, resistance to meropenem reached 33.3% in this cohort. This finding implies that empirical escalation to meropenem for suspected sepsis may be ineffective in approximately one-third of cases in this unit. The high meropenem resistance was predominantly driven by K. *pneumoniae* (accounting for 60% of carbapenem-resistant isolates), followed by S. *marcescens* (20%) and A. *baumannii* (20%). While carbapenem resistance has historically been uncommon in neonatal settings, recent Jordanian data indicate an emerging trend, with reported meropenem resistance rates ranging from 10% to over 25% among Gram-negative neonatal isolates, particularly in late-onset and multidrug-resistant infections [20, 25, 46].

Resistance was also observed in non-β-lactam agents, including trimethoprim-sulfamethoxazole (53.1%) and ciprofloxacin (25.7%), further narrowing available therapeutic options.

Several factors likely contribute to and favor the emergence of multidrug-resistant organisms and the substantial resistance burden observed in this cohort. According to national studies, key contributors include antimicrobial overuse and repeated exposure to broad-spectrum agents such as carbapenems and vancomycin, along with prolonged NICU hospitalization and cumulative exposure to invasive devices [25, 46]. In addition, limited availability of structured antimicrobial stewardship programs and NICU-specific antibiograms has been highlighted as a challenge in both Jordanian and regional neonatal care settings [20, 49].

## Conclusion

The study findings reinforce the persistent, predominant role of multidrug-resistant Gram-negative pathogens in neonatal sepsis at a Jordanian tertiary NICU, consistent with national and regional trends. The sustained burden over the last decade, along with emerging, alarming resistance to last-line agents such as carbapenems, underscores the limited impact of current control measures. The risk factors identified in our cohort, including prematurity and invasive interventions align with those reported in the literature, highlighting their reproducibility across settings.

These findings emphasize the urgent need for data-driven antibiotic stewardship, including the development and application of NICU-specific antibiograms to guide timely and effective empirical therapy while minimizing unnecessary antibiotic exposure. Addressing the substantial burden of Gram-negative neonatal sepsis requires a coordinated, interdisciplinary approach that combines enhanced infection prevention, active national surveillance, and early risk stratification.

### Strengths

This is the first cohort-based national study to investigate risk factors, bacterial profiles, and antimicrobial resistance of Gram-negative neonatal sepsis, addressing a critical gap in national evidence. Our analysis included a large, well-characterized dataset of 4,172 blood culture samples from 3,429 neonates over six years, which assisted with a thorough assessment of Gram-negative sepsis. The use of standardized clinical and laboratory definitions, along with the systematic classification of cases by pathogen type and stratification by onset timing, enabled clinically meaningful analyses. This statistical approach, which included multivariable regression and sensitivity analyses, accounted for confounders and strengthened the validity of the study results. Finally, the real-world clinical setting enhances the external validity and applicability of the study results.

### Limitations

This study has several limitations. First, it was conducted at a single center, which may limit generalizability. Second, although the overall cohort was large, the EOS subgroup included only 9 cases, limiting statistical power; thus, EOS-specific findings should be considered descriptive and interpreted with caution. Third, the timing of some clinical interventions relative to the onset of clinical suspicion and definitive diagnosis of sepsis could not be determined, as variables were recorded retrospectively rather than at standardized time points. Fourth, residual confounding by baseline illness may remain, as the retrospective design limits full adjustment between groups. Fifth, our regression identifies factors associated with Gram-negative sepsis versus no sepsis, rather than factors uniquely associated with Gram-negative etiology. A direct comparison between Gram-negative and Gram-positive sepsis would be required to achieve etiological specificity. Sixth, the analysis primarily focused on neonatal factors, and detailed maternal data -such as prolonged rupture of membranes, chorioamnionitis, and intrapartum antibiotic exposure- were unavailable, restricting comprehensive risk assessment, particularly for EOS, and should be considered when interpreting EOS-related findings. Seventh, blood culture was used as the unit of analysis (4,804 cultures from 3,429 neonates); some neonates contributed multiple cultures, including repeat cultures within the same Gram-negative sepsis episode or non-sepsis admissions. Although 78.5% contributed only one culture, residual within-neonate correlation cannot be excluded. Inclusion of multiple cultures may violate the independence assumption of standard logistic regression and slightly underestimate standard errors with narrower confidence intervals; however, given the low frequency of repeated cultures, the impact on effect size estimates is likely limited. Future studies should consider mixed-effects models or cluster-robust standard errors to account for within-neonate clustering and provide more precise inference. Eighth, the low number of culture-negative sepsis cases reflects the strict definition requiring both clinical and treatment criteria and possible under-documentation in electronic records; prospective studies may capture higher prevalence. Ninth, although confirmed infections were excluded from the non-sepsis group, the retrospective design precludes complete certainty that all infections were documented, and some comparison episodes may have included undiagnosed infections.

## Supplementary Information

Below is the link to the electronic supplementary material.

Supplementary Material 1

Supplementary Material 2

Supplementary Material 3

Supplementary Material 4

Supplementary Material 5

## Data Availability

The datasets generated and analyzed during the current study are not publicly available due to ethical restrictions, as they contain detailed clinical information that could compromise participant confidentiality. The data are available from the corresponding author upon reasonable request and with approval from the Jordan University Hospital Institutional Review Board. A data transfer agreement may be required, and all uses must comply with the terms of the original ethical approval (IRB Approval No. 10/2023/16360).

## Abbreviations

aOR: Adjusted Odds Ratio
CI: Confidence Interval
CL: Central Line
CLABSI: Central Line-Associated Bloodstream Infection
CPR: Cardio Pulmonary Resuscitation
CRP: C-reactive protein
CVC: Central Venous Catheter
EHR: Electronic Health Records
ELBW: extremely low birth weight
EOS: Early Onset Sepsis
ESBL: Extended Spectrum Beta Lactamase
FCS: Fully Conditional Specification
JUH: Jordan University Hospital
LMIC: Low and Middle-Income Countries
LOS: Late Onset Sepsis
MDR: Multi Drug Resistance
NICU: Neonatal Intensive Care Unit
NS: Neonatal Sepsis
OR: Odds Ratio
PCT: Procalcitonin test
PLT: Platelet
ROP: Retinopathy of Prematurity
TNF-α: Tumor Necrosis Factor-Alpha
TPN: Total Parenteral Nutrition
VLBW: Very low birth weight

## References

[1] Birrie E, Sisay E, Tibebu NS, Tefera BD, Zeleke M, Tefera Z. Neonatal sepsis and associated factors among newborns in Woldia and Dessie Comprehensive Specialized Hospitals, North-East Ethiopia, 2021. Infect Drug Resist. 2022;15:4169–79. 10.2147/IDR.S374835. 35937781 10.2147/IDR.S374835 PMC9354861

[2] World Health Organization. Newborns: improving survival and well-being [Internet]. Geneva: World Health Organization. 2020 [cited 2025 Dec 27]. Available from: https://www.who.int/news-room/fact-sheets/detail/newborns-reducing-mortality.

[3] Fleischmann C, Reichert F, Cassini A, Horner R, Harder T, Markwart R, et al. Global incidence and mortality of neonatal sepsis: a systematic review and meta-analysis. Arch Dis Child. 2021;106(8):745–52. 10.1136/archdischild-2020-320217. 33483376 10.1136/archdischild-2020-320217 PMC8311109

[4] Odabasi IO, Bulbul A. Neonatal sepsis. Sisli Etfal Hastan Tip Bul. 2020;54(2):142–58. 10.14744/SEMB.2020.00236. 32617051 10.14744/SEMB.2020.00236 PMC7326682

[5] Stocker M, Mangeret Fuerst R, Agyeman PKA, McDougall J, Berger C, Giannoni E. Management of neonates at risk of early onset sepsis: a probability based approach and recent literature appraisal: update of the Swiss national guideline of the Swiss Society of Neonatology and the Pediatric Infectious Disease Group Switzerland. Eur J Pediatr. 2024;183(12):5517–29. 10.1007/s00431-024-05811-0. 39417838 10.1007/s00431-024-05811-0 PMC11527939

[6] Attia Hussein Mahmoud H, Parekh R, Dhandibhotla S, Sai T, Pradhan A, Alugula S, et al. Insight into neonatal sepsis: an overview. Cureus. 2023;15(9):e45530. 10.7759/cureus.45530. 37868444 10.7759/cureus.45530 PMC10585949

[7] Raturi A, Chandran S. Neonatal sepsis: aetiology, pathophysiology, diagnostic advances and management strategies. Clin Med Insights Pediatr. 2024;18:11795565241281337. 10.1177/11795565241281337. 39371316 10.1177/11795565241281337 PMC11452898

[8] Ershad M, Mostafa A, Dela Cruz M, Vearrier D. Neonatal sepsis. Curr Emerg Hosp Med Rep. 2019;7(3):83–90. 10.1007/s40138-019-00188-z. 32226657 10.1007/s40138-019-00188-z PMC7100521

[9] Tang A, Shi Y, Dong Q, Wang Z, Zhu X, Liu F, et al. Prognostic differences in sepsis caused by Gram-negative bacteria and Gram-positive bacteria: a systematic review and meta-analysis. Crit Care. 2023;27(1):467. 10.1186/s13054-023-04750-w. 38037118 10.1186/s13054-023-04750-w PMC10691150

[10] Wen SCH, Ezure Y, Rolley L, Spurling G, Lau CL, Riaz S, et al. Gram-negative neonatal sepsis in low- and lower-middle-income countries and WHO empirical antibiotic recommendations: a systematic review and meta-analysis. PLoS Med. 2021;18(9):e1003787. 10.1371/journal.pmed.1003787. 34582466 10.1371/journal.pmed.1003787 PMC8478175

[11] Kaiser Permanente neonatal early onset sepsis calculator [Internet]. Kaiser Permanente Division of Research; c2024 [cited 2026 Jan 15]. Available from: https://neonatalsepsiscalculator.kaiserpermanente.org.

[12] Magiorakos AP, Srinivasan A, Carey RB, Carmeli Y, Falagas ME, Giske CG, et al. Multidrug-resistant, extensively drug-resistant and pandrug-resistant bacteria: an international expert proposal for interim standard definitions for acquired resistance. Clin Microbiol Infect. 2012;18(3):268–81. 10.1111/j.1469-0691.2011.03570.x. 21793988 10.1111/j.1469-0691.2011.03570.x

[13] Clinical and Laboratory Standards Institute. Performance standards for antimicrobial susceptibility testing. CLSI supplement M100. 2026;36th ed:402–p.Clinical and Laboratory Standards InstituteWayne (PA).

[14] Manandhar S, Amatya P, Ansari I, Joshi N, Shah S, Shrestha S, et al. Risk factors for the development of neonatal sepsis in a neonatal intensive care unit of a tertiary care hospital of Nepal. BMC Infect Dis. 2021;21:546. 10.1186/s12879-021-06261-x. 34107906 10.1186/s12879-021-06261-x PMC8191200

[15] Wynn JL, Wong HR. Pathophysiology of neonatal sepsis. In: Polin RA, Abman SH, Rowitch DH, Benitz WE, editors. Fetal and neonatal physiology. 5th ed. Philadelphia: Elsevier; 2017. pp. 1536–e5210.

[16] Stocker M, van Herk W, El Helou S, Dutta S, Schuerman FABA, van den Tooren-de Groot RK, et al. C-reactive protein, procalcitonin, and white blood count to rule out neonatal early-onset sepsis within 36 hours: a secondary analysis of the Neonatal Procalcitonin Intervention Study. Clin Infect Dis. 2021;73(2):e383–90. 10.1093/cid/ciaa876. 32881994 10.1093/cid/ciaa876

[17] Yin W, Fang C, Fan X, Chen Y. Albumin and C-reactive protein as diagnostic markers for neonatal sepsis: a retrospective study. J Int Med Res. 2024;52(3):03000605241238993. 10.1177/03000605241238993. 38530043 10.1177/03000605241238993 PMC10966997

[18] Jin Y, Guo S, Xiao Y, Yin C. Assessment of the diagnostic significance of pentraxin-3 in conjunction with procalcitonin (PCT) and C-reactive protein (CRP) for neonatal sepsis. BMC Infect Dis. 2025;25(1):401. 10.1186/s12879-025-10821-w. 40128667 10.1186/s12879-025-10821-w PMC11934617

[19] Ree IMC, Fustolo-Gunnink SF, Bekker V, Fijnvandraat KJ, Steggerda SJ, Lopriore E. Thrombocytopenia in neonatal sepsis: incidence, severity and risk factors. PLoS ONE. 2017;12(10):e0185581. 10.1371/journal.pone.0185581. 28977011 10.1371/journal.pone.0185581 PMC5627935

[20] Alameri M, Gharaibeh L, Alsous M, Yaghi A, Tanash A, Sa’id S, et al. Antibiotic prescription practice and resistance patterns of bacterial isolates from a neonatal intensive care unit: a retrospective study from Jordan. Antibiot (Basel). 2025;14(1):105. 10.3390/antibiotics14010105. 10.3390/antibiotics14010105 PMC11762691 39858390

[21] Centers for Disease Control and Prevention. Describing epidemiologic data. Principles of epidemiology in public health practice. 3rd ed. Atlanta (GA): US Department of Health and Human Services, CDC; 2021. pp. 3–1.

[22] Clinical and Laboratory Standards Institute. Analysis and presentation of cumulative antimicrobial susceptibility test data. 5th ed. CLSI guideline M39. Wayne (PA): Clinical and Laboratory Standards Institute. 2022. 88 p.

[23] Sterne JAC, White IR, Carlin JB, Spratt M, Royston P, Kenward MG, et al. Multiple imputation for missing data in epidemiological and clinical research: potential and pitfalls. BMJ. 2009;338:b2393. 10.1136/bmj.b2393. 19564179 10.1136/bmj.b2393 PMC2714692

[24] Abdelfattah R, Al Shboul O, Al Ali M, Fakhoury R. Bacterial prevalence and inflammatory changes in positive blood culture in community-acquired neonatal sepsis in Jordan. Int J Infect Dis Reg. 2025;17:100756. 10.1016/j.ijregi.2024.100756. 10.1016/j.ijregi.2025.100756 PMC12513297 41081039

[25] Al Lawama M, Badran E, Khuri Bulos N. Neonatal Gram-negative sepsis in a tertiary hospital in Jordan: when fever means multidrug resistance! Pediatr Ther. 2014;4(4):212. 10.4172/2161-0665.1000212.

[26] Milton R, Gillespie D, Dyer C, Taiyari K, Carvalho MJ, Thomson K, et al. Neonatal sepsis and mortality in low-income and middle-income countries from a facility-based birth cohort: an international multisite prospective observational study. Lancet Glob Health. 2022;10(5):e661–72. 10.1016/S2214-109X(22)00043-2. 35427523 10.1016/S2214-109X(22)00043-2 PMC9023753

[27] Hallmaier-Wacker LK, Andrews A, Nsonwu O, Lamont G, Ladhani S, Hope R, et al. Incidence and aetiology of infant Gram-negative bacteraemia and meningitis: systematic review and meta-analysis. Arch Dis Child. 2022;107(3):e1–8. 10.1136/archdischild-2021-322862. 35710719 10.1136/archdischild-2022-324047 PMC9606543

[28] Huynh BT, Kermorvant-Duchemin E, Chheang R, Randrianirina F, Seck A, Hariniaina R, et al. Severe bacterial neonatal infections in Madagascar, Senegal, and Cambodia: a multicentric community-based cohort study. PLoS Med. 2021;18(10):e1003792. 10.1371/journal.pmed.1003792. 34582450 10.1371/journal.pmed.1003681 PMC8478182

[29] Klinger G, Levy I, Sirota L, Boyko V, Reichman B, Lerner-Geva L, et al. Late-onset sepsis among extremely preterm infants of 24–28 weeks’ gestation: international trends, mortality, and length of stay. Neonatology. 2024;121(6):761–70. 10.1159/000539578. 38889700 10.1159/000539245 PMC11633898

[30] Coggins SA, Glaser K. Updates in late-onset neonatal sepsis: risk assessment, prevention, and outcomes. NeoReviews. 2022;23(11):e756–70. 10.1542/neo.23-11-e756. 10.1542/neo.23-10-e738 PMC9675597 36316254

[31] Al Dasoky HA, Al Awaysheh FN, Kaplan NM, Al Rimawi HA, Agha RM, Abu-Setteh MH. Risk factors for neonatal sepsis in a tertiary hospital in Jordan. J R Med Serv. 2009;16(3):16–9.

[32] Khassawneh M, Khader Y, Abuqtaish N. Clinical features of neonatal sepsis caused by resistant Gram-negative bacteria. Pediatr Int. 2009;51(3):332–6. 10.1111/j.1442-200X.2008.02767.x. 19400829 10.1111/j.1442-200X.2008.02767.x

[33] Wang X, Tang K, Chen L, Cheng S, Xu H. Association between neonatal sepsis and retinopathy of prematurity: a systematic review and meta-analysis. BMJ Open. 2019;9(9):e025440. 10.1136/bmjopen-2018-025440. 31129577 10.1136/bmjopen-2018-025440 PMC6537987

[34] Glaser K, Poindexter BB, Adams-Chapman I, Bann CM, Hintz SR, Cotten CM, et al. Neonatal sepsis episodes and risk of retinopathy of prematurity in very preterm infants. JAMA Netw Open. 2024;7(3):e2423933. 10.1001/jamanetworkopen.2024.23933. 39052290 10.1001/jamanetworkopen.2024.23933 PMC11273231

[35] Hapsari AT, Krisniawati N, Syiraz TA, Pratidina RWG. Association between prematurity and neonatal sepsis: a case-control study at a tertiary referral hospital in Indonesia. Med Health J. 2025;5(1):45–53. 10.20884/1.mhj.2025.5.1.17292.

[36] Murthy S, Godinho MA, Guddattu V, Lewis L, Nair NS. Risk factors of neonatal sepsis in India: a systematic review and meta-analysis. PLoS ONE. 2019;14(4):e0215683. 10.1371/journal.pone.0215683. 31022223 10.1371/journal.pone.0215683 PMC6483350

[37] Bech CM, Myrnerts Höök S, Nalwadda G, Onyango D, Wamani H, Retsä I, et al. Risk factors for neonatal sepsis in Sub-Saharan Africa: a systematic review with meta-analysis. BMJ Open. 2022;12(9):e054491. 10.1136/bmjopen-2021-054491. 36253895 10.1136/bmjopen-2021-054491 PMC9438195

[38] Hoffman A, Satyavolu S, Muhanna D, Malay S, Raffay T, Windau A, et al. Predictors of mortality and severe illness from Escherichia coli sepsis in neonates. J Perinatol. 2024;44(12):1816–21. 10.1038/s41372-024-02117-9. 39266664 10.1038/s41372-024-02117-9 PMC11606913

[39] Garg PM, Paschal JL, Ansari MAY, Adams K, Jilling T, Kandasamy J. Clinical impact of NEC-associated sepsis on outcomes in preterm infants. Pediatr Res. 2022;91(4):802–9. 10.1038/s41390-021-01613-4. 10.1038/s41390-022-02034-7 PMC10311923 35352003

[40] Shane AL, Sánchez PJ, Stoll BJ. Neonatal sepsis. Lancet. 2017;390(10104):1770–80. 10.1016/S0140-6736(17)31002-4. 28434651 10.1016/S0140-6736(17)31002-4

[41] Johnson J, Malwade S, Agarkhedkar S, Randive B, Rajput UC, Valvi C, et al. Risk factors for health care-associated bloodstream infections in NICUs. JAMA Netw Open. 2025;8(3):e251821. 10.1001/jamanetworkopen.2025.1821. 40131271 10.1001/jamanetworkopen.2025.1821 PMC11937935

[42] Shin J, Kang HM, Kim SY, Youn YA, Choi CW, Chang YS. The effect of minimizing central line days for very low birth weight infants through quality improvement. Sci Rep. 2024;14(1):3854. 10.1038/s41598-024-53163-4. 38360733 10.1038/s41598-024-53163-4 PMC10869738

[43] Stevens TP, Schulman J. Evidence-based approach to preventing central line-associated bloodstream infection in the NICU. Acta Paediatr. 2012;101(464):11–6. 10.1111/j.1651-2227.2011.02547.x. 22404886 10.1111/j.1651-2227.2011.02547.x

[44] Auriti C, Ronchetti MP, Pezzotti P, Marrocco G, Quondamcarlo A, Seganti G, et al. Determinants of nosocomial infection in 6 neonatal intensive care units: an Italian multicenter prospective cohort study. Infect Control Hosp Epidemiol. 2010;31(9):926–33. 10.1086/655461. 20645863 10.1086/655461

[45] West BA, Peterside O, Ugwu RO, Eneh AU. Prospective evaluation of the usefulness of C-reactive protein in the diagnosis of neonatal sepsis in a sub-Saharan African region. Antimicrob Resist Infect Control. 2012;1(1):22. 10.1186/2047-2994-1-22. 22958461 10.1186/2047-2994-1-22 PMC3436619

[46] Yusef D, Shalakhti T, Awad S, Algharaibeh H, Khasawneh W. Clinical characteristics and epidemiology of sepsis in the neonatal intensive care unit in the era of multi-drug resistant organisms: a retrospective review. Pediatr Neonatol. 2018;59(1):35–41. 10.1016/j.pedneo.2017.06.005. 28642139 10.1016/j.pedneo.2017.06.001

[47] Almudeer AH, Alibrahim MA, Gosadi IM. Epidemiology and risk factors associated with early onset neonatal sepsis in the south of KSA. J Taibah Univ Med Sci. 2020;15(6):509–14. 10.1016/j.jtumed.2020.09.002. 33318743 10.1016/j.jtumed.2020.08.009 PMC7715412

[48] Hammoud MS, Al-Taiar A, Al-Abdi SY, Bozaid H, Khan A, AlMuhairi LM, et al. Late-onset neonatal sepsis in Arab states in the Gulf region: two-year prospective study. Int J Infect Dis. 2017;55:125–30. 10.1016/j.ijid.2016.12.024. 28088587 10.1016/j.ijid.2017.01.006

[49] Al Dabbagh M, Alghounaim M, Almaghrabi R, Al-Rashid M, AlShahrani D, Alshehri M, et al. Healthcare-associated Gram-negative infections among pediatric patients in Middle Eastern countries: a narrative review. Infect Dis Ther. 2023;12(10):2225–52. 10.1007/s40121-023-00872-4. 10.1007/s40121-023-00799-w PMC10229515 37071349

