# Supplementary material for: Burden, risk factors, and emerging microbiological trends of Gram-negative neonatal sepsis in Jordan: a retrospective cohort study
Source: BMC Infect Dis. 2026 May 18;26:1312. doi: 10.1186/s12879-026-13529-7 (PMC13366917; doi:10.1186/s12879-026-13529-7)
Supplement: Supplementary file 1 — Supplementary Material 1 [file 12879_2026_13529_MOESM1_ESM.docx]

**Additional File 1.** Definitions of Variables Used in the Analysis

| Variable | Definition | Categories | Reference |
| --- | --- | --- | --- |
| Gender | Biological sex of the neonate as recorded at birth | Male  Female | - |
| Gestational age (weeks) | Gestational maturity at birth calculated in completed weeks | Extremely preterm: neonate born at <28 weeks gestation  Very preterm: neonate born between 28^+0^- and 32^+6^-weeks gestation  Moderate to late preterm: neonate born between 33^+0^- and 36^+6^-weeks gestation  Term: Neonate born at or over 37 weeks of gestation | [4,5] |
| Mode of delivery | Method of childbirth | Vaginal  Cesarean | - |
| Birth weight categories (BW) (g) | Neonatal birth weight classification | Extremely low BW (ELBW): neonate weight at birth <1000 g  Very low BW (VLBW): neonate weight at birth 1000-1499 g  Low BW (LBW): neonate weight at birth 1500-2499 g  Normal BW (NBW): neonate weight at birth 2500-3999 g  High BW (HBW): neonate weight at birth 4000-4500 g  Very high BW (VHBW): neonate weight at birth >4500 g | [4,10] |
| Apgar score at 1 minute | Apgar score assessed at 1 minute after birth | Severe distress ≤3  Moderate difficulty 4–6  Assuring ≥7 | [10] |
| Apgar score at 5 minutes | Apgar score assessed at 5 minutes after birth | Severe distress ≤3  Moderate difficulty 4–6  Assuring ≥7 | [10] |
| C-reactive protein (CRP) (mg/L) | Serum CRP level measured as an inflammatory marker | Normal <10 mg/L  Increased 10–100 mg/L  Markedly increased >100 mg/L | [16-18] |
| Hemoglobin (HB) (g/dL) | Hemoglobin concentration in peripheral blood | Anemia <13.5 g/dL  Normal ≥13.5 g/dL | [13] |
| White blood cell count (WBC) (×10⁹/L) | Total leukocyte count in peripheral blood | Leukopenia <5 ×10⁹/L  Normal 5–25 ×10⁹/L  Leukocytosis >25 ×10⁹/L | [4,8] |
| Absolute neutrophil count (ANC) (×10⁹/L) | Absolute neutrophil count calculated from differential count | Neutropenia <1.5 ×10⁹/L  No neutropenia >1.5 ×10⁹/L | [14,15] |
| Absolute lymphocyte count (ALC) (×10⁹/L) | Absolute lymphocyte count calculated from differential count | Lymphopenia <2 ×10⁹/L  No lymphopenia >2 ×10⁹/L | [15] |
| Platelet count (×10⁹/L) | Platelet concentration in peripheral blood | Thrombocytopenia <150 ×10⁹/L  Normal 150–450 ×10⁹/L  Thrombocytosis >450 ×10⁹/L | [18,19] |
| Feeding type | Primary type of feeding during hospitalization | Mother’s milk  Formula  Mixed feeding (Mother’s milk + formula)  Donor milk  Mother and donor milk  Formula and donor milk | - |
| Umbilical venous catheter (UVC) insertion | Placement of an umbilical venous catheter during hospitalization | No UVC  UVC | - |
| Central line (CL) insertion | Placement of any central venous catheter other than UVC; PICC (peripherally Inserted Central Catheter) or BROVIAK during hospitalization | No CL  CL | - |
| Timing of CL insertion relative to blood culture | Timing of CL insertion in relation to blood culture sampling | Before culture  After culture | - |
| Intubation | Requirement for endotracheal intubation during hospitalization | No intubation  Intubation | - |
| Timing of intubation relative to blood culture | Timing of intubation in relation to blood culture sampling | Before culture  After culture | - |
| Blood transfusion | Administration of any blood product during hospitalization | No  Yes | - |
| Surgery done | Performance of any surgical procedure during hospitalization | No  Yes | - |
| Chest tube insertion | Placement of a chest tube during hospitalization | No chest tube  Chest tube | - |
| Abdominal paracentesis | Performance of abdominal paracentesis during hospitalization | No  Yes | - |
| Cardiopulmonary resuscitation (CPR) | Performance of CPR at any time during hospital stay | No  Yes | - |
| Retinopathy of prematurity (ROP) | Diagnosis of ROP during hospitalization | No  Yes | - |
| Death | In-hospital mortality status | No  Yes | - |
| Length of stay | Duration of hospital stay in days | - | - |
| Age at CL insertion (days) | Postnatal age in days at time of CL insertion | - | - |
| Age at intubation (days) | Postnatal age in days at time of intubation | - | - |
| Multi Drug Resistance (MDR) | Resistance to at least one antimicrobial agent in three or more distinct antibiotic categories | No  Yes | [13] |

# References

1. Birrie E, Sisay E, Tibebu NS, Tefera BD, Zeleke M, Tefera Z. Neonatal sepsis and associated factors among newborns in Woldia and Dessie Comprehensive Specialized Hospitals, North-East Ethiopia, 2021. Infect Drug Resist. 2022;15:4169-79. doi:10.2147/IDR.S374835.
2. World Health Organization. Newborns: improving survival and well-being [Internet]. Geneva: World Health Organization; 2020 [cited 2025 Dec 27]. Available from: <https://www.who.int/news-room/fact-sheets/detail/newborns-reducing-mortality>
3. Fleischmann C, Reichert F, Cassini A, Horner R, Harder T, Markwart R, et al. Global incidence and mortality of neonatal sepsis: a systematic review and meta-analysis. Arch Dis Child. 2021;106(8):745-52. doi:10.1136/archdischild-2020-320217.
4. Odabasi IO, Bulbul A. Neonatal sepsis. Sisli Etfal Hastan Tip Bul. 2020;54(2):142-58. doi:10.14744/SEMB.2020.00236.
5. Stocker M, Mangeret Fuerst R, Agyeman PKA, McDougall J, Berger C, Giannoni E. Management of neonates at risk of early onset sepsis: a probability based approach and recent literature appraisal: update of the Swiss national guideline of the Swiss Society of Neonatology and the Pediatric Infectious Disease Group Switzerland. Eur J Pediatr. 2024;183(12):5517-29. doi:10.1007/s00431-024-05811-0.
6. Attia Hussein Mahmoud H, Parekh R, Dhandibhotla S, Sai T, Pradhan A, Alugula S, et al. Insight into neonatal sepsis: an overview. Cureus. 2023;15(9):e45530. doi:10.7759/cureus.45530.
7. Raturi A, Chandran S. Neonatal sepsis: aetiology, pathophysiology, diagnostic advances and management strategies. Clin Med Insights Pediatr. 2024;18:11795565241281337. doi:10.1177/11795565241281337.
8. Ershad M, Mostafa A, Dela Cruz M, Vearrier D. Neonatal sepsis. Curr Emerg Hosp Med Rep. 2019;7(3):83-90. doi:10.1007/s40138-019-00188-z.
9. Tang A, Shi Y, Dong Q, Wang Z, Zhu X, Liu F, et al. Prognostic differences in sepsis caused by Gram-negative bacteria and Gram-positive bacteria: a systematic review and meta-analysis. Crit Care. 2023;27(1):467. doi:10.1186/s13054-023-04750-w.
10. Wen SCH, Ezure Y, Rolley L, Spurling G, Lau CL, Riaz S, et al. Gram-negative neonatal sepsis in low- and lower-middle-income countries and WHO empirical antibiotic recommendations: a systematic review and meta-analysis. PLoS Med. 2021;18(9):e1003787. doi:10.1371/journal.pmed.1003787.
11. Kaiser Permanente neonatal early onset sepsis calculator [Internet]. Kaiser Permanente Division of Research; c2024 [cited 2026 Jan 15]. Available from: [https://neonatalsepsiscalculator.kaiserpermanente.org](https://neonatalsepsiscalculator.kaiserpermanente.org/)
12. Magiorakos AP, Srinivasan A, Carey RB, Carmeli Y, Falagas ME, Giske CG, et al. Multidrug-resistant, extensively drug-resistant and pandrug-resistant bacteria: an international expert proposal for interim standard definitions for acquired resistance. Clin Microbiol Infect. 2012;18(3):268-81. doi:10.1111/j.1469-0691.2011.03570.x.
13. Clinical and Laboratory Standards Institute. Performance standards for antimicrobial susceptibility testing. 36th ed. CLSI supplement M100. Wayne (PA): Clinical and Laboratory Standards Institute; 2026. 402 p.
14. Manandhar S, Amatya P, Ansari I, Joshi N, Shah S, Shrestha S, et al. Risk factors for the development of neonatal sepsis in a neonatal intensive care unit of a tertiary care hospital of Nepal. BMC Infect Dis. 2021;21:546. doi:10.1186/s12879-021-06261-x.
15. Wynn JL, Wong HR. Pathophysiology of neonatal sepsis. In: Polin RA, Abman SH, Rowitch DH, Benitz WE, editors. Fetal and neonatal physiology. 5th ed. Philadelphia: Elsevier; 2017. p. 1536-52.e10.
16. Stocker M, van Herk W, El Helou S, Dutta S, Schuerman FABA, van den Tooren-de Groot RK, et al. C-reactive protein, procalcitonin, and white blood count to rule out neonatal early-onset sepsis within 36 hours: a secondary analysis of the Neonatal Procalcitonin Intervention Study. Clin Infect Dis. 2021;73(2):e383-e390. doi:10.1093/cid/ciaa876.
17. Yin W, Fang C, Fan X, Chen Y. Albumin and C-reactive protein as diagnostic markers for neonatal sepsis: a retrospective study. J Int Med Res. 2024;52(3):03000605241238993. doi:10.1177/03000605241238993.
18. Jin Y, Guo S, Xiao Y, Yin C. Assessment of the diagnostic significance of pentraxin-3 in conjunction with procalcitonin (PCT) and C-reactive protein (CRP) for neonatal sepsis. BMC Infect Dis. 2025;25(1):401. doi:10.1186/s12879-025-10821-w.
19. Ree IMC, Fustolo-Gunnink SF, Bekker V, Fijnvandraat KJ, Steggerda SJ, Lopriore E. Thrombocytopenia in neonatal sepsis: incidence, severity and risk factors. PLoS One. 2017;12(10):e0185581. doi:10.1371/journal.pone.0185581.
20. Alameri M, Gharaibeh L, Alsous M, Yaghi A, Tanash A, Sa'id S, et al. Antibiotic prescription practice and resistance patterns of bacterial isolates from a neonatal intensive care unit: a retrospective study from Jordan. Antibiotics (Basel). 2025;14(1):105. doi:10.3390/antibiotics14010105.
21. Centers for Disease Control and Prevention. Describing epidemiologic data. In: Principles of epidemiology in public health practice. 3rd ed. Atlanta (GA): US Department of Health and Human Services, CDC; 2021. p. 3-1–3-28.
22. Clinical and Laboratory Standards Institute. Analysis and presentation of cumulative antimicrobial susceptibility test data. 5th ed. CLSI guideline M39. Wayne (PA): Clinical and Laboratory Standards Institute; 2022. 88 p.
23. Sterne JAC, White IR, Carlin JB, Spratt M, Royston P, Kenward MG, et al. Multiple imputation for missing data in epidemiological and clinical research: potential and pitfalls. BMJ. 2009;338:b2393. doi:10.1136/bmj.b2393.
24. Abdelfattah R, Al Shboul O, Al Ali M, Fakhoury R. Bacterial prevalence and inflammatory changes in positive blood culture in community-acquired neonatal sepsis in Jordan. Int J Infect Dis Regions. 2025;17:100756. doi:10.1016/j.ijregi.2024.100756.
25. Al Lawama M, Badran E, Khuri Bulos N. Neonatal Gram-negative sepsis in a tertiary hospital in Jordan: when fever means multidrug resistance! Pediatr Ther. 2014;4(4):212. doi:10.4172/2161-0665.1000212.
26. Milton R, Gillespie D, Dyer C, Taiyari K, Carvalho MJ, Thomson K, et al. Neonatal sepsis and mortality in low-income and middle-income countries from a facility-based birth cohort: an international multisite prospective observational study. Lancet Glob Health. 2022;10(5):e661-e672. doi:10.1016/S2214-109X(22)00043-2.
27. Hallmaier-Wacker LK, Andrews A, Nsonwu O, Lamont G, Ladhani S, Hope R, et al. Incidence and aetiology of infant Gram-negative bacteraemia and meningitis: systematic review and meta-analysis. Arch Dis Child. 2022;107(3):e1-e8. doi:10.1136/archdischild-2021-322862.
28. Huynh BT, Kermorvant-Duchemin E, Chheang R, Randrianirina F, Seck A, Hariniaina R, et al. Severe bacterial neonatal infections in Madagascar, Senegal, and Cambodia: a multicentric community-based cohort study. PLoS Med. 2021;18(10):e1003792. doi:10.1371/journal.pmed.1003792.
29. Klinger G, Levy I, Sirota L, Boyko V, Reichman B, Lerner-Geva L, et al. Late-onset sepsis among extremely preterm infants of 24-28 weeks' gestation: international trends, mortality, and length of stay. Neonatology. 2024;121(6):761-70. doi:10.1159/000539578.
30. Coggins SA, Glaser K. Updates in late-onset neonatal sepsis: risk assessment, prevention, and outcomes. NeoReviews. 2022;23(11):e756-e770. doi:10.1542/neo.23-11-e756.
31. Al Dasoky HA, Al Awaysheh FN, Kaplan NM, Al Rimawi HA, Agha RM, Abu-Setteh MH. Risk factors for neonatal sepsis in a tertiary hospital in Jordan. J R Med Serv. 2009;16(3):16-9.
32. Khassawneh M, Khader Y, Abuqtaish N. Clinical features of neonatal sepsis caused by resistant Gram-negative bacteria. Pediatr Int. 2009;51(3):332-6. doi:10.1111/j.1442-200X.2008.02767.x.
33. Wang X, Tang K, Chen L, Cheng S, Xu H. Association between neonatal sepsis and retinopathy of prematurity: a systematic review and meta-analysis. BMJ Open. 2019;9(9):e025440. doi:10.1136/bmjopen-2018-025440.
34. Glaser K, Poindexter BB, Adams-Chapman I, Bann CM, Hintz SR, Cotten CM, et al. Neonatal sepsis episodes and risk of retinopathy of prematurity in very preterm infants. JAMA Netw Open. 2024;7(3):e2423933. doi:10.1001/jamanetworkopen.2024.23933.
35. Hapsari AT, Krisniawati N, Syiraz TA, Pratidina RWG. Association between prematurity and neonatal sepsis: a case-control study at a tertiary referral hospital in Indonesia. Med Health J. 2025;5(1):45-53. doi:10.20884/1.mhj.2025.5.1.17292.
36. Murthy S, Godinho MA, Guddattu V, Lewis L, Nair NS. Risk factors of neonatal sepsis in India: a systematic review and meta-analysis. PLoS One. 2019;14(4):e0215683. doi:10.1371/journal.pone.0215683.
37. Bech CM, Myrnerts Höök S, Nalwadda G, Onyango D, Wamani H, Retsä I, et al. Risk factors for neonatal sepsis in Sub-Saharan Africa: a systematic review with meta-analysis. BMJ Open. 2022;12(9):e054491. doi:10.1136/bmjopen-2021-054491.
38. Hoffman A, Satyavolu S, Muhanna D, Malay S, Raffay T, Windau A, et al. Predictors of mortality and severe illness from Escherichia coli sepsis in neonates. J Perinatol. 2024;44(12):1816-21. doi:10.1038/s41372-024-02117-9.
39. Garg PM, Paschal JL, Ansari MAY, Adams K, Jilling T, Kandasamy J. Clinical impact of NEC-associated sepsis on outcomes in preterm infants. Pediatr Res. 2022;91(4):802-9. doi:10.1038/s41390-021-01613-4.
40. Shane AL, Sánchez PJ, Stoll BJ. Neonatal sepsis. Lancet. 2017;390(10104):1770-80. doi:10.1016/S0140-6736(17)31002-4.
41. Johnson J, Malwade S, Agarkhedkar S, Randive B, Rajput UC, Valvi C, et al. Risk factors for health care-associated bloodstream infections in NICUs. JAMA Netw Open. 2025;8(3):e251821. doi:10.1001/jamanetworkopen.2025.1821.
42. Shin J, Kang HM, Kim SY, Youn YA, Choi CW, Chang YS. The effect of minimizing central line days for very low birth weight infants through quality improvement. Sci Rep. 2024;14(1):3854. doi:10.1038/s41598-024-53163-4.
43. Stevens TP, Schulman J. Evidence-based approach to preventing central line-associated bloodstream infection in the NICU. Acta Paediatr. 2012;101(464):11-6. doi:10.1111/j.1651-2227.2011.02547.x.
44. Auriti C, Ronchetti MP, Pezzotti P, Marrocco G, Quondamcarlo A, Seganti G, et al. Determinants of nosocomial infection in 6 neonatal intensive care units: an Italian multicenter prospective cohort study. Infect Control Hosp Epidemiol. 2010;31(9):926-33. doi:10.1086/655461.
45. West BA, Peterside O, Ugwu RO, Eneh AU. Prospective evaluation of the usefulness of C-reactive protein in the diagnosis of neonatal sepsis in a sub-Saharan African region. Antimicrob Resist Infect Control. 2012;1(1):22. doi:10.1186/2047-2994-1-22.
46. Yusef D, Shalakhti T, Awad S, Algharaibeh H, Khasawneh W. Clinical characteristics and epidemiology of sepsis in the neonatal intensive care unit in the era of multi-drug resistant organisms: a retrospective review. Pediatr Neonatol. 2018;59(1):35-41. doi:10.1016/j.pedneo.2017.06.005.
47. Almudeer AH, Alibrahim MA, Gosadi IM. Epidemiology and risk factors associated with early onset neonatal sepsis in the south of KSA. J Taibah Univ Med Sci. 2020;15(6):509-14. doi:10.1016/j.jtumed.2020.09.002.
48. Hammoud MS, Al-Taiar A, Al-Abdi SY, Bozaid H, Khan A, AlMuhairi LM, et al. Late-onset neonatal sepsis in Arab states in the Gulf region: two-year prospective study. Int J Infect Dis. 2017;55:125-30. doi:10.1016/j.ijid.2016.12.024.
49. Al Dabbagh M, Alghounaim M, Almaghrabi R, Al-Rashid M, AlShahrani D, Alshehri M, et al. Healthcare-associated Gram-negative infections among pediatric patients in Middle Eastern countries: a narrative review. Infect Dis Ther. 2023;12(10):2225-52. doi:10.1007/s40121-023-00872-4.
