# Supplementary material for: Burden, risk factors, and emerging microbiological trends of Gram-negative neonatal sepsis in Jordan: a retrospective cohort study
Source: BMC Infect Dis. 2026 May 18;26:1312. doi: 10.1186/s12879-026-13529-7 (PMC13366917; doi:10.1186/s12879-026-13529-7)
Supplement: Supplementary file 2 — Supplementary Material 2 [file 12879_2026_13529_MOESM2_ESM.docx]

**Additional File 2.** Pooled multivariable logistic regression results for factors associated with neonatal gram-negative sepsis (N* = 4,172) (Sepsis vs. No sepsis).

| **Variable** | **Category (Reference)** | **OR** | **95% CI** | **P value** |
| --- | --- | --- | --- | --- |
| **Demographic And Perinatal Characteristics** | | | | |
| **Gender** | Male vs Female | 1.309 | 0.660-2.598 | 0.441 |
| **Gestational Age** | Extremely preterm vs Term | 0.185 | 0.051-0.674 | **0.011** |
|  | Very preterm vs Term | 3.104 | 0.775-12.424 | 0.110 |
|  | Moderate–late preterm vs Term | 1.618 | 0.593-4.420 | 0.348 |
| **Birth Weight*** | ELBW vs Normal | Could not estimate | | 0.999 |
|  | VLBW vs Normal | Could not estimate | | 0.999 |
|  | LBW vs Normal | Could not estimate | | 0.999 |
|  | HBW vs Normal | Could not estimate | | 0.999 |
|  | VHBW vs Normal | Could not estimate | | 0.999 |
| **Mode Of Delivery** | Cesarean vs Vaginal | 1.426 | 0.629-3.237 | 0.396 |
| **Apgar Score at 1 Minute** | Distressed vs Normal | 2.135 | 0.035-128.475 | 0.714 |
|  | Moderate vs Normal | 3.038 | 0.035-262.062 | 0.621 |
| **Apgar Score at 5 Minutes** | Distressed vs Normal | 0.457 | 0.039-5.303 | 0.530 |
|  | Moderate vs Normal | 0.263 | 0.006-11.709 | 0.489 |
| **Laboratory Parameters** | | | | |
| **C-Reactive Protein (CRP)** | Increased vs Normal | 14.808 | 6.415-34.192 | **<0.001** |
|  | markedly increased vs Normal | 174.208 | 47.603-637.536 | **<0.001** |
| **Hemoglobin (HB)** | Anemia vs Normal | 0.441 | 0.190-1.023 | 0.057 |
| **WBC Count (WBC)** | Leukopenia vs Normal | 0.496 | 0.089-2.776 | 0.425 |
|  | Leukocytosis vs Normal | 0.803 | 0.092-7.044 | 0.843 |
| **Absolute Neutrophil Count (ANC)** | Neutropenia vs Normal | 0.453 | 0.053-3.848 | 0.468 |
| **Absolute Lymphocyte Count (ALC)** | Lymphopenia vs Normal | 2.453 | 0.912-6.597 | 0.075 |
| **Platelet Count (Plt)*** | Thrombocytopenia vs Normal | 2.564 | 1.252-5.253 | **0.010** |
|  | Thrombocytosis vs Normal | 0.242 | 0.029-2.019 | 0.190 |
| **Clinical Interventions** | | | | |
| **Umbilical Venous Catheter (Uvc) Insertion** | Inserted vs Not inserted | 0.997 | 0.465-2.140 | 0.995 |
| **Surgery Performed** | Yes vs No | 1.240 | 0.506-3.037 | 0.638 |
| **Chest Tube Insertion*** | Inserted vs Not inserted | Could not estimate | | 0.996 |
| **Abdominal Paracentesis*** | Done vs Not done | Could not estimate | | 1.000 |
| **Blood Transfusion** | Yes vs No | 0.586 | 0.218-1.572 | 0.288 |
| **Feeding Type*** | Formula vs Mother’s milk | Could not estimate | | 1.000 |
|  | Mixed feeding (Mother’s milk + formula) vs Mother’s milk | Could not estimate | | 1.000 |
|  | Donor milk vs Mother’s milk | Could not estimate | | 1.000 |
|  | Mother and donor milk vs Mother’s milk | Could not estimate | | 1.000 |
|  | Formula and donor milk vs Mother’s milk | Could not estimate | | 1.000 |
| **Timing of CL Insertion Relative to Blood Culture**** | After vs Before culture | 0.51 | 0.35-0.76  0.35-0.76 | <0.001 |
| **Age at CL Insertion (Days)**** | Per 1-day increase | 0.999 | 0.99-1.01 | 0.906 |
| **Timing of Intubation Relative to Blood Culture**** | After vs Before culture | 0.39 | 0.28-0.53 | <0.001 |
| **Age at Intubation (Days)**** | Per 1-day increase | 1.00 | 0.999-1.003 | 0.319 |
| **NICU Length of Stay (Days)** | Per 1-day increase | 0.997 | 0.980-1.015 | 0.736 |

***Not estimable due to extremely sparse data**

****Analyzed only in the subset of neonates who received the intervention (central line: n = 114; intubation: n = 682).**

**Pooled estimates are from 20 multiply imputed datasets combined using Rubin’s rules; odds ratios, confidence intervals, and p-values reflect these pooled results.**
