# Supplementary material for: Burden, risk factors, and emerging microbiological trends of Gram-negative neonatal sepsis in Jordan: a retrospective cohort study
Source: BMC Infect Dis. 2026 May 18;26:1312. doi: 10.1186/s12879-026-13529-7 (PMC13366917; doi:10.1186/s12879-026-13529-7)
Supplement: Supplementary file 3 — Supplementary Material 3 [file 12879_2026_13529_MOESM3_ESM.docx]

**Additional File 3.** Missing Values and Imputation Approach for Study Variables

| **Characteristic** | **Count** | **# of missing values** | **Percentage**  **%** | **Imputation** |
| --- | --- | --- | --- | --- |
| **Gender** | 4172 | 0 | .0 | None |
| **Gestational age (weeks)** | 4030 | 142 | 3.4 | None |
| **Mode of delivery** | 4172 | 0 | .0 | None |
| **Birth weight (BW) (g)** | 4168 | 4 | .1 | None |
| **Apgar Score at 1 minute** | 3723 | 449 | 10.8 | Imputation: 20  Imputation Method: Fully Conditional specification (MCMC), maximum iterations= 10  Model type for scale variables: Linear Regression |
| **Apgar Score at 5 minutes** | 3720 | 452 | 10.8 | Imputation: 20  Imputation Method: Fully Conditional specification (MCMC), maximum iterations= 10  Model type for scale variables: Linear Regression |
| **C-reactive protein (CRP) (mg/L)** | 3999 | 173 | 4.1 | None |
| **Hemoglobin (HB) (g/dL)** | 4012 | 160 | 3.8 | None |
| **White Blood Cell Count (WBC) (×10⁹/L)** | 4012 | 160 | 3.8 | None |
| **Absolute Neutrophil Count (ANC) (×10⁹/L)** | 4012 | 160 | 3.8 | None |
| **Absolute Lymphocyte Count (ALC) (×10⁹/L)** | 4012 | 160 | 3.8 | None |
| **Platelet Count (×10⁹/L)** | 4004 | 168 | 4.0 | None |
| **Feeding Type** | 3473 | 699 | 16.8 | Imputation: 20  Imputation Method: Fully Conditional specification (MCMC), maximum iterations= 10  Model type for scale variables: Linear Regression |
| **Umbilical Venous Catheter (UVC) Insertion** | 4172 | 0 | .0 | None |
| **Central Line (CL) Insertion** | 4172 | 0 | .0 | None |
| **Timing of CL Insertion Relative to Blood Culture (Only participants who had a CL inserted)** | 114 | 0 | .0 | None |
| **Intubation** | 4172 | 0 | .0 | None |
| **Timing of Intubation Relative to Blood Culture (Only participants who were intubated)** | 679 | 3 | 0.4 | None |
| **Blood Transfusion** | 4172 | 0 | .0 | None |
| **Surgery Done** | 4172 | 0 | .0 | None |
| **Chest Tube Insertion** | 4172 | 0 | .0 | None |
| **Abdominal Paracentesis** | 4172 | 0 | .0 | None |
| **Cardiopulmonary Resuscitation (CPR)** | 4172 | 0 | .0 | None |
| **Retinopathy of Prematurity (ROP)** | 4172 | 0 | .0 | None |
| **Death** | 4172 | 0 | .0 | None |
| **Length of Stay** | 4171 | 1 | .0 | None |
| **Age at CL insertion (days)**  **(Only participants who had a CL inserted)** | 114 | 0 | .0 | None |
| **Age at Intubation (days)**  **(Only participants who were intubated)** | 679 | 3 | 0.4 | None |
