# Supplementary material for: Burden, risk factors, and emerging microbiological trends of Gram-negative neonatal sepsis in Jordan: a retrospective cohort study
Source: BMC Infect Dis. 2026 May 18;26:1312. doi: 10.1186/s12879-026-13529-7 (PMC13366917; doi:10.1186/s12879-026-13529-7)
Supplement: Supplementary file 4 — Supplementary Material 4 [file 12879_2026_13529_MOESM4_ESM.docx]

**Additional file 4.** Performance Metrics of Logistic Regression Models Across the Full Cohort and Defined Subsets

| **Table:** Logistic Regression Model Performance for Full Cohort and Subsets | | | | | | | |
| --- | --- | --- | --- | --- | --- | --- | --- |
| **Model / Subset** | **Hosmer & Lemeshow χ² (df)** | **p-value** | **Omnibus χ² (df)** | **p-value** | **-2 Log Likelihood** | **Cox & Snell R²** | **Nagelkerke R²** |
| Full cohort | 7.41 (8) | 0.49 | 239.82 (37) | <0.001 | 323.77 | 0.063 | 0.444 |
| Central line placement | 5.09 (7) | 0.65 | 0.71 (2) | 0.70 | 57.22 | 0.01 | 0.02 |
| Intubation | 1.60 (3) | 0.66 | 2.07 (2) | 0.36 | 249.86 | 0.00 | 0.01 |
