# Supplementary material for: Burden, risk factors, and emerging microbiological trends of Gram-negative neonatal sepsis in Jordan: a retrospective cohort study
Source: BMC Infect Dis. 2026 May 18;26:1312. doi: 10.1186/s12879-026-13529-7 (PMC13366917; doi:10.1186/s12879-026-13529-7)
Supplement: Supplementary file 5 — Supplementary Material 5 [file 12879_2026_13529_MOESM5_ESM.docx]

**Additional File 5.** Sensitivity Analyses of Gestational Age and Platelet Abnormalities Adjusted for Clinical Interventions

1. **Gestational Age**

| **Table 1.** Sensitivity Analysis: Gestational Age and Sepsis Adjusted for Clinical Interventions | | | |
| --- | --- | --- | --- |
| **Predictor (Category)** | **aOR** | **95% CI** | **p-value** |
| Extremely preterm vs Term | 6.89 | 5.49-8.65 | <0.001 |
| Very preterm vs Term* | 5.30 | 4.33-6.49 | <0.001 |
| Moderate-late preterm vs Term* | 2.97 | 2.42-3.64 | <0.001 |
| Central line placement (Yes vs No) | 1.88 | 1.58-2.25 | <0.001 |
| Intubation (Yes vs No) | 2.31 | 2.05-2.60 | <0.001 |
| Surgery (Yes vs No) | 1.90 | 1.67-2.17 | <0.001 |
| Blood transfusion (Yes vs No) | 1.70 | 1.48-1.97 | <0.001 |
| Chest tube (Yes vs No) | - | - | - |
| ***In the unadjusted analysis the association with Gram-negative sepsis was not statistically significant** | | | |

1. **Platelet Abnormalities**

| **Table 2.** Sensitivity Analysis: Platelet Abnormalities and Sepsis Adjusted for Clinical Interventions | | | |
| --- | --- | --- | --- |
| **Predictor (Category)** | **aOR** | **95% CI** | **p-value** |
| Thrombocytopenia vs Normal | 4.37 | 3.91-4.89 | <0.001 |
| Thrombocytosis vs Normal* | 0.26 | 0.17-0.40 | <0.001 |
| Central line placement (Yes vs No) | 2.17 | 1.80-2.61 | <0.001 |
| Intubation (Yes vs No) | 1.95 | 1.72-2.21 | <0.001 |
| Surgery (Yes vs No) | 1.08 | 0.93-1.25 | 0.323 |
| Blood transfusion (Yes vs No) | 2.62 | 2.30-2.99 | <0.001 |
| Chest tube (Yes vs No) | - | - | - |
| ***In the unadjusted analysis the association with Gram-negative sepsis was not statistically significant** | | | |
